# GM-CSF impairs erythropoiesis by disrupting erythroblastic island formation via macrophages

**DOI:** 10.1186/s12967-021-03214-5

**Published:** 2022-01-03

**Authors:** Weijie Cao, Wenjuan Fan, Fang Wang, Yinyin Zhang, Guanghua Wu, Xiaojing Shi, Jian xiang Shi, Fengcai Gao, Meimei Yan, Rong Guo, Yingmei Li, Wei Li, Chunyan Du, Zhongxing Jiang

**Affiliations:** 1 Department of Hematology, The First Affiliated Hospital of Zhengzhou University, Zhengzhou, 450052 Henan China; 2 The Academy of Medical Science, College of Medical, Zhengzhou University, Zhengzhou, 450052 Henan China; 3 Laboratory Animal Center, School of Medical Sciences, Zhengzhou University, Zhengzhou, 450052 Henan China; 4 BGI College & Henan Institute of Medical and Pharmaceutical Sciences in Academy of Medical Science, Zhengzhou University, Zhengzhou, 450052 Henan China; 5 Department of Hematology, The Affiliated Cancer Hospital of Zhengzhou University, Zhengzhou, 450008 Henan China

**Keywords:** GMCSF, EBI macrophages, Erythropoiesis, Anemia of inflammatory diseases

## Abstract

Anemia is a significant complication of chronic inflammation and may be related to dysregulated activities among erythroblastic island (EBI) macrophages. GM-CSF was reported to be upregulated and attracted as a therapeutic target in many inflammatory diseases. Among EBIs, we found that the GM-CSF receptor is preferentially and highly expressed among EBI macrophages but not among erythroblasts. GM-CSF treatment significantly decreases human EBI formation in vitro by decreasing the adhesion molecule expression of CD163. RNA-sequence analysis suggests that GM-CSF treatment impairs the supporting function of human EBI macrophages during erythropoiesis. GM-CSF treatment also polarizes human EBI macrophages from M2-like type to M1-like type. In addition, GM-CSF decreases mouse bone marrow (BM) erythroblasts as well as EBI macrophages, leading to a reduction in EBI numbers. In defining the molecular mechanism at work, we found that GM-CSF treatment significantly decreases the adhesion molecule expression of CD163 and Vcam1 in vivo. Importantly, GM-CSF treatment also decreases the phagocytosis rate of EBI macrophages in mouse BM as well as decreases the expression of the engulfment-related molecules Mertk, Axl, and Timd4. In addition, GM-CSF treatment polarizes mouse BM EBI macrophages from M2-like type to M1-like type. Thus, we document that GM-CSF impairs EBI formation in mice and humans. Our findings support that targeting GM-CSF or reprogramming EBI macrophages might be a novel strategy to treat anemia resulting from inflammatory diseases.

**Supplementary Information:**

The online version contains supplementary material available at 10.1186/s12967-021-03214-5.

## Key points

- GM-CSF impairs human EBI formation by decreasing CD163 adhesion molecule expression.
- GM-CSF decreases mouse BM erythroblasts and EBI macrophages as well as impairs EBI formation via decreasing surface expression of CD163 and Vcam1.

## Introduction

Granulocyte–macrophage colony-stimulating factor (GM-CSF), a significant myelopoietic growth factor and pro-inflammatory cytokine, has been shown to be upregulated and has attracted increasing interest as a therapeutic target for many inflammatory diseases, including coronavirus disease 2019 (COVID-19; [1–3]). In general, GM-CSF is barely detectable in the peripheral blood of healthy people, and GM-CSF plays a minor role in homeostatic myelopoiesis, as evidenced by the fact that GM-CSF knock-out mice have a virtually normal lifespan and have less dramatic alterations in the basal myeloid system [4, 5]. However, severe GM-CSF deficiency causes pulmonary alveolar proteinosis (PAP), a life-threatening interstitial lung disease in which dysfunctional alveolar macrophages cannot clear surfactant [6, 7]. For erythropoiesis, human GM-CSF stimulates primitive and definitive erythropoiesis in mouse embryos expressing human GM-CSF receptors [8]. Additionally, GM-CSF levels increased in sickle cell disease (SCD), leading to downregulation of fetal hemoglobin expression [9]. Importantly, interleukin-6 (IL-6), interferon-γ (IFN-γ) and GM-CSF were notably unregulated in the mouse model of anemia of inflammation (AI) induced by heat-killed Brucella abortus [10, 11]. Despite these studies, the role of GM-CSF per se in adult human and mouse erythropoiesis remains unclear.

Erythropoiesis is a process by which hematopoietic stem cells (HSCs) proliferate and differentiate via multiple distinct developmental stages, to eventually generate mature red blood cells (RBCs; [12–14]). The process occurs at the erythroblastic island (EBI), which is composed of a central macrophage surrounded by developing erythroid cells [15]. The EBI, first described by *Marcel Bessis* in 1958 [15], was functionally validated by Narla Mohandas et al. [16], who found significantly lower numbers in hyper-transfused rat bone marrow (BM; [16]). Recently, studies indicated that EBI macrophages promote erythropoiesis by directly interacting with erythroblasts, secreting growth factors, phagocytosing senescent RBCs, providing iron, and finally engulfing the nucleus enucleated by erythroblasts [13, 17, 18]. Many hematopoietic growth factors regulate erythropoiesis by affecting the function of EBI macrophages. Erythropoietin (EPO) acts on both erythroid cells and EBI macrophages simultaneously to ensure efficient erythropoiesis [17, 19]; Granulocyte colony-stimulating factor (G-CSF) blocks medullary erythropoiesis by depleting EBI macrophages in mouse BM [20, 21]. These functional studies strongly suggest that hematopoietic growth factors can regulate erythropoiesis by affecting the roles of EBI macrophages.

In previous studies, both G-CSF and GM-CSF were used to mobilize HSCs from the BM into the blood in order to harvest large quantities of HSCs for subsequent transplantation in humans [22, 23]. Both G-CSF and GM-CSF cause HSCs mobilization by perturbing HSC niches in the BM. To define these mechanisms, studies have shown that G-CSF results in downregulation of the cell adhesion molecule vascular cell adhesion molecule-1(VCAM-1) and the chemokine CXC-motif ligand-12(CXCL12; [24, 25]). These two molecules are both essential to HSC retention within the BM. Additionally, studies have also shown that the effect of G-CSF on HSC niches is mediated in part by a subpopulation of BM macrophages [26]. G-CSF also causes a significant loss of BM macrophages expressing VCAM-1, CD169, and ER-HR3 and blocks medullary erythropoiesis in BM [20]. Furthermore, using imaging flowcytometry (IFC), *Joshua Tay *et al*.* found that G-CSF reduces EBI frequency in the BM by more than 100 times [21]. In contrast to the many studies of G-CSF’s role in regulating erythropoiesis, the precise role of GM-CSF is less well understood.

In the present study, we demonstrate that GM-CSF significantly decreases human EBI formation in vitro. Bioinformatics analysis of RNA sequencing (RNA-seq) on GM-CSF-treated and control EBI macrophages further confirmed the impaired EBI formation, as evidenced by decreased adhesion molecule expression of CD163. In particular, GM-CSF injection into mice also significantly decreases BM erythroblasts as well as EBI numbers by decreasing both the number of EBI macrophages and adhesion molecule expression of CD163 and of Vcam1. Our study presents novel data that GM-CSF impairs erythropoiesis by disturbing EBI formation and proposes targeting EBI macrophages as a potential treatment option for AI.

## Materials and methods

### Antibodies and mice

All antibodies related to this study are listed in Additional file 9: Table S1. Wild type (WT) mice that are 8–12 weeks old are on the C57BL/6 background and are maintained at the Experimental Animal Center of Zhengzhou University. GM-CSF (300 μg/kg, Do-D1 or D0-D3) was injected into sex-matched male and female mice to study the functional role in vivo. Clodronate-loaded liposomes injection experiment was performed as previous described [27].

### Blood parameter analysis

About 30–50 μl of peripheral blood was collected through the orbital vein using an Eppendorf tube containing 1 μl of 0.5 M ethylenediaminetetraacetate (EDTA; Fisher) after anesthesia. Blood was diluted 1:10 in phosphate balanced saline (PBS) and analyzed via an advia120 hematology analyzer. The RBC numbers, hemoglobin, hematocrit (HCT), and reticulocytes of mice before and after GM-CSF injection were analyzed using GraphPad Prism 9.0 software.

### Preparation of single cells for flowcytometry

Under terminal anesthesia using isoflurane, mice were killed via cervical dislocation. The BM and spleen (SP) were collected and processed for single-cell preparation for flowcytometry. The single cell suspensions were prepared as previously described [17]. In brief, BM cells were flushed with PBS + 2%FBS + 2 mM EDTA, and SP cells were smashed in PBS + 2%FBS + 2 mM EDTA. Then, the cell suspensions were washed with PBS plus 2% fetal bovine serum (FBS) and 2 mM EDTA, centrifugated at 300 g for 10 min and gently pushed through a 70 µm cell strainer. The single cells suspension was then used for flowcytometry staining and analyses.

### Flowcytometry staining and analyses

For burst-forming unit erythroid (BFU-E) and colony-forming unit erythroid (CFU-E) staining, 5 × 10^6^ cells were blocked with 50 μL PBS + 0.5%BSA containing rat anti-mouse CD16/CD32 (dilution 1:100) for 15 min at 4℃, then stained with BV-421-Lin (1μL/5 × 10^6^cells), BV-421-CD41(1μL/5 × 10^6^cells), PE-Cy7-CD34(1μL/5 × 10^6^cells), PE-Cy7-CD16/32(1μL/5 × 10^6^ cells), APC-Cy7-Scal1(1μL/5 × 10^6^ cells), APC-CD117(1μL/5 × 10^6^ cells), and Percp-CD71(1μL/5 × 10^6^ cells) for 30 min on ice in the dark. For murine erythroblast staining, 3 × 10^6^cells were blocked with 25 μL PBS + 0.5%BSA containing rat anti-mouse CD16/CD32 (dilution 1:100) for 15 min at 4℃, then stained with anti-CD11b-APC-Cy7 (0.1 µg/10^6^cells), anti-Gr1-APC-Cy7 (0.1 µg/10^6^cells), anti-CD45-APC-Cy7 (0.1 µg/10^6^cells), anti-Ter119-PE (0.5 µg/10^6^cells) or FITC-Ter119(0.5 µg/10^6^cells), and anti-CD44-APC (0.2 µg/10^6^cells) for 30 min on ice in the dark. For macrophage staining, 5 × 10^6^ cells were blocked with 50 μL PBS + 0.5%BSA containing rat anti-mouse CD16/CD32 (dilution 1:100) for 15 min at 4℃, then stained with anti-CD11b-APC-Cy7 (0.1 µg/10^6^cells), anti-Gr1-APC-Cy7 (0.1 µg/10^6^cells), anti-AF647-F4/80 (5 µg/10^6^ cells), anti-Percpcy5.5-CD106 (0.3 µg/10^6^ cells), anti-FITC-CD169 (4 µg/10^6^ cells), anti-Percp-CD163 (0.2 µg/10^6^ cells), anti-FITC-Timd4 (0.25 µg/10^6^ cells), anti-PE-Cy7-Mertk (0.5 µg/10^6^ cells), anti-PE-Cy7Axl (0.125 µg/10^6^ cells), anti-PE-Cy7-MHC-II (0.5μL/5 × 10^6^ cells), anti-FITC-CD206 (1μL/5 × 10^6^ cells), anti-Percpcy5.5-CD14 (1μL/5 × 10^6^ cells), anti-FITC-CD86 (1μL/5 × 10^6^ cells), and anti-PE-Cy7-CD80 (1μL/5 × 10^6^ cells) for 30 min on ice in the dark. After staining, the cells were washed once with PBS plus 0.5%BSA and 2 mM EDTA. DAPI were used to gate out dead cells. Then, cells were resuspended with PBS plus 0.5%BSA and 2 mM EDTA, and run on a BD Air III (BD bioscience). Flow Jo software (BD) was used to analyze the data.

### P-STAT5 staining

For P-STAT5 staining, the BD Transcription Factor Phospho Buffer Set (BD Cat#565575) was used. In short, the BM cells were starved for 4 h, and were stimulated with GM-CSF for 15 min. Then, the cells were fixed with TF Fix/Perm Buffer(1X) for 50 min at 4℃ and washed using 1 × TFP Perm/Wash Buffer. After fixation, cells were incubated with BD Phosflow™ Perm Buffer III, and the cells were washed once to remove Perm Buffer III using 1 × TFP Perm/Wash Buffer. Finally, cells were stained with CD11b, Gr-1, F4/80, and P-stat5 (ThermoFisher Scientific,12–9010-42) for 50 min on ice in the dark, washed using 1 × TFP Perm/Wash Buffer, and run on a BD Air III. Flow Jo software (BD) was used to analyze the data.

### Engulfment staining and analysis

We followed the same method as *Jessica A. Hamerman’s* group [28]. BM single cells from both control and GM-CSF treatment mice were washed and prepared for intracellular staining with Fixation and Permeabilization buffer (BD Biosciences), washed in Perm/Wash buffer (BD Biosciences); and then stained with anti-CD11b-APC-Cy7 (0.1 µg/10^6^cells), anti-Gr1-APC-Cy7 (0.1 µg/10^6^cells), anti-AF647-F4/80 (5 µg/10^6^ cells), and anti-FITC-Ter119 (0.5 µg/10^6^cells) for 30 min on ice in the dark to detect cells that had phagocytosed RBCs. BD Air III was used to collect events and Flow Jo software (BD) was used to analyze the data.

### EBI enrichment

EBIs in mouse BM were enriched and the numbers were quantified. Our EBI enrichment method matches that of previous studies [17, 21, 29]. The protocol is as follows: (1) preparation of different concentration of density gradient solution (0%, 1.5% and 3%, Additional file 10: TableS2). (2) flush all of 4 bones (2 femur and 2 tibia) in 2 mL of 0% density gradient solution using a 1 mL syringe with a 25 G (tibia) or 23 G (femur) needle in doses of 500 μL (rapidly in order to preserve erythroblastic islands). (3) gently pipette the bone cells flushed with a 1 ml syringe (once or twice only) and filter through a 70 μm cell strainer. (4) In a 50 ml centrifuge tube first add 5 mL of 3% density gradient solution, then slowly add 5 mL of 1.5% density gradient solution along the side wall of the tube with a pipette to bring it above the 3% density gradient solution. A further 5 mL of 0% density gradient solution was slowly added with a pipette along the side wall of the tube to place it above the 1.5% density gradient solution. The bone marrow cell solution containing the erythroblastic islands was adjusted to a volume of 5 mL with 0% density gradient solution and slowly added to the tube containing the stratified density gradient solution with a pipette and placed on top of the 0% density gradient solution. (5) leave at room temperature for 30 min. Pipette the 0% and 1.5% density layers into a new 15 mL tube., and collect the 3% layer, which contains the erythroblastic islands. (6) Cytospins were performed using 3% layer from mouse BM, and the number of EBIs in each slide was quantified.

### Frozen section preparation and hematoxylin and eosin (HE) staining

SP cells were obtained from both GM-CSF treated and control mice and immediately embedded into an optimal cutting temperature (OCT) compound. The tissues were kept in a − 80 ℃ freezer. Then, hematoxylin and eosin (HE) staining was performed as previously described [30].

### Co-culture of human “EBI-like” macrophages with late stages of erythroblasts

Erythroblasts and “EBI-like” macrophages were derived from cord blood CD34^+^ cells. The detailed cultured method was same as our previously described [12, 14, 17, 31]. Fresh CD34^+^ cells were purified by CD34 MicroBeads (Miltenyi Biotec, Gladbach, Germany) from human healthy donors at Zhengzhou University. The cell culture procedure was comprised of 3 phases. In the present, two phases of cell culture were used. Composition of the base culture medium was Iscove's Modified Dulbecco's Medium (IMDM, Invitrogen), 2% human peripheral blood plasma (Stem Cell Technologies), 3% human AB serum (Atlanta Biologicals), 200 μg/mL Holo-human transferrin (Sigma Aldrich), 3 IU/mL heparin (The First Affiliated Hospital of Zhengzhou University), and 10 μg/mL insulin (The First Affiliated Hospital of Zhengzhou University). In the first phase (day 0 to day 6), CD34^+^ cells at a concentration of 10^5^/mL were cultured in the presence of 10 ng/mL stem cell factor (SCF, Stem Cell Technologies), 1 ng/mL IL-3(Stem Cell Technologies), and 3 IU/mL erythropoietin (The First Affiliated Hospital of Zhengzhou University). In the second phase (day 7 to day 11), IL-3 was omitted from the culture medium. Then, the D11 erythroblasts were harvested for the next step of co-culture experiment. Human “EBI-like” macrophages were also derived from CD34^+^ cells. In the Day 0 to Day 7, CD34^+^ cells were cultured in IMDM containing 2% human peripheral blood plasma, 3% human AB serum, 3 IU/mL heparin, 10 µg/mL insulin, 10 ng/mL SCF, 1 ng/mL IL-3, 100 ng/mL M-CSF and 50 ng/mL FLT3, 1 × penicillin–streptomycin. At day 7, the suspensions were removed and IMDM containing 2% human peripheral blood plasma, 3% human AB serum, 3 IU/mL heparin, 10 µg/mL insulin, 100 ng/mL M-CSF and 50 ng/mL FLT3 were added. Then, the adherent cells were cultured for another 4 days. At D11, all the adherent cells will differentiate into macrophages as our previously described [17]. Macrophages were pretreated with 100 ng/mL GM-CSF for 24 h. Untreated or GM-CSF-pretreated macrophages were mixed with day 11 erythroblasts at a 1: 20 ratio and cultured for 12 h in an IMDM medium containing 2% human peripheral blood plasma, 3% human AB serum, 3 IU/mL heparin, 10 µg/mL insulin, 200 µg/mL holo-human transferrin, 10 IU/ml EPO, 5 mM Mg^2+^, and 5 mM Ca^2+^. 1 × 10^5^ cells were collected for cytospin analysis.

### RNA-sequencing

RNA-seq was prepared and analyzed as previously described [14, 17, 31, 32]. RNA was extracted from control and GM-CSF treated “EBI-like” macrophages. Approximately 100 ng of total RNA was used as input for cDNA library preparation, which was preformed using an Illumina TruSeq kit followed by sequencing using an Illumina HiSeq 4000 platform (Beijing Genomics Institute, BGI, China). For analysis of the RNA-seq data, gene read counts for gencode hg19 version 31 protein coding genes were generated using kallisto [33]. Differential gene expression was examined with DESeq2 using a Wald test, where the log2 fold change is greater than 0.5 with a 0.05 adjusted p-value cutoff and independent filtering to remove genes with lower expression levels, as previously described [12]. Gene set enrichment analysis (GSEA) was performed as previously described [34]. The permutation number was set to 1000 and the permutation type was set to gene set. The raw data was uploaded to the National Omics Data Encyclopedia (NODE) database (https://www.biosino.org/node) under accession number OEP002596.

### Cell count

Absolute cell count was measured via flowcytometry using 123count eBeads™ Counting Beads (Cat#: 01–1234-42) according to the manufacturer’s protocol.

### Enrichment of BM F4/80^+^ macrophages

Control and GM-CSF-treated mouse BM F4/80^+^ macrophages were enriched via F4/80 microbeads (Miltenyi, Cat#:130–110-443). BM cells were incubated with F4/80 microbeads for 15 min on ice in the dark. The cells were washed with PBS plus 2% FBS and 2 mM EDTA, centrifugated at 300 g for 10 min, and resuspended with 2 mL PBS plus 2% FBS and 2 mM EDTA. Finally, the BM F4/80^+^ macrophages were enriched by Quadro MACS following the manufacturer's instructions. More than 90% purification was achieved, as previously described [35]. These cells were used for QRT-PCR.

### QRT-PCR, cytospins and Giemsa-Wright staining

QRT-PCR, cytospins and Giemsa-Wright staining were performed as in our previous papers [14, 17, 31, 32]. Primers of human *CD163, CD169, EMP* and *αV-integrin* used in the present study are the same in our previous work [17]. The primers of mouse *IL1-β, TNF-α, iNOS, TGF-β, IL-10* and *Arg1* are listed in Additional file 11: Table S3.

### Statistics

GraphPad Prism 9.0 software (GraphPad Software, Inc.) was used to perform the statistical analysis. All the experiments were replicated at least three times. All data were reported as mean ± SEM. Comparisons between different groups were performed by Student’s t test. *P* < 0.05 was considered to indicate a statistically significant difference.

## Results

### GM-CSF treatment leads to impaired human EBI formation by decreasing adhesion molecule expression of CD163

**Fig. 1:**
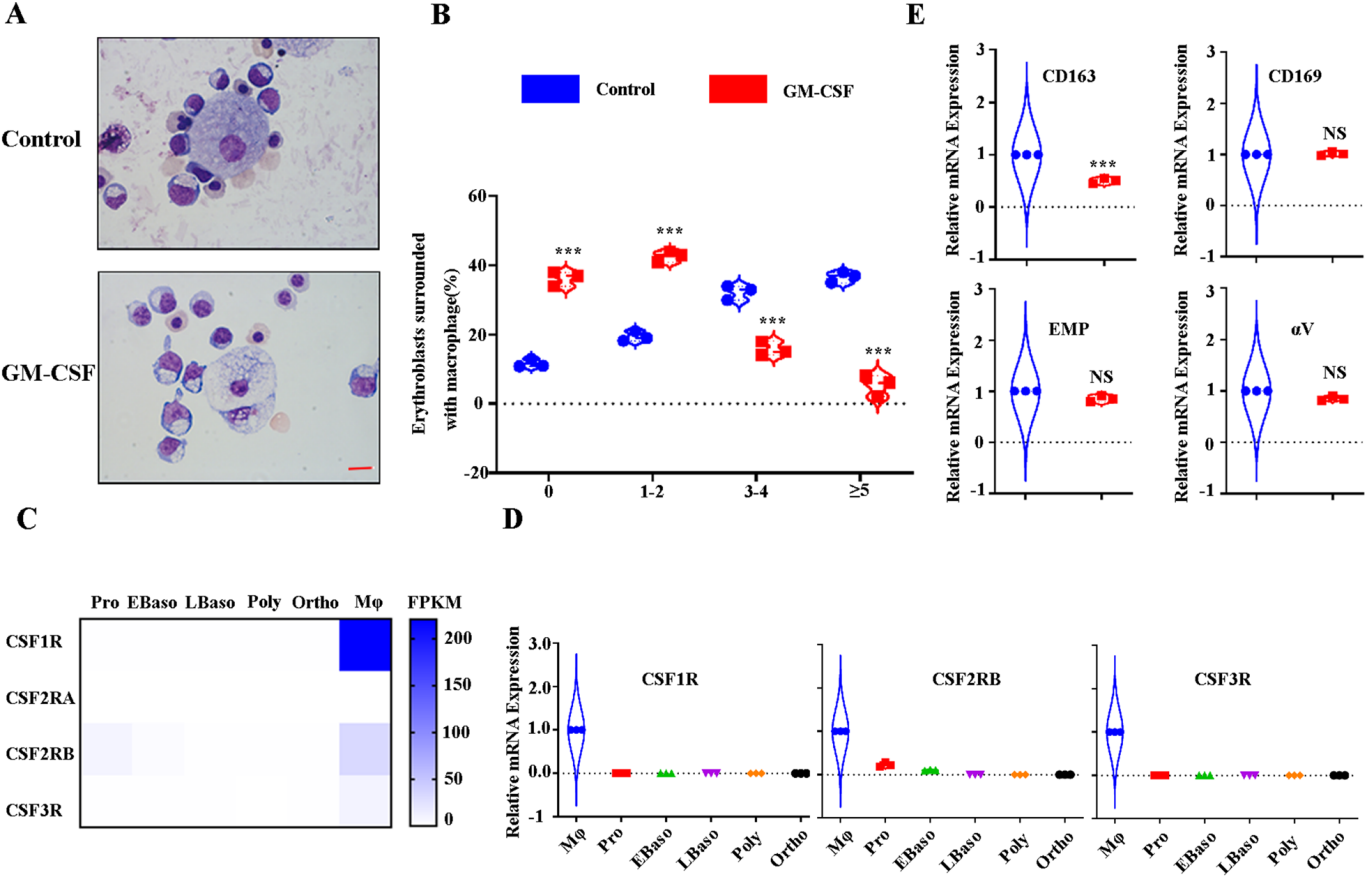
GM-CSF treatment leads to impaired human EBI formation by decreasing CD163 expression. **A** Representative cytospin image of EBI formed between late-stage erythroblasts and control and GM-CSF pretreatment macrophages in vitro. **B** Quantitative analysis of the percentage of EBIs associated with different erythroblast numbers (0,1–2,3–4, ≥ 5). **C** Heatmap showing mRNA levels of CSF1R, CSF2RA, CSF2RB, and CSF3R as assessed by RNA-seq in distinct stages of erythroblasts and EBI macrophages. **D** QRT-PCR results showing mRNA levels of CSF1R, CSF2RA, CSF2RB, and CSF3R in distinct stages of erythroblasts and EBI macrophages. **E** QRT-PCR results showing mRNA levels of CD163, CD169, MAEA, and αV-integrin of control as well as GM-CSF pretreatment macrophages in vitro. *P < 0.05, ** *P* < 0.01 *** *P* < 0.001. N = 3

To begin to study the potential roles of GM-CSF on human EBI formation, we performed human EBI formation assay using “EBI-like” macrophages and late-stage erythroblasts derived from human cord blood CD34^+^ cells as previously described [17]. “EBI-like” macrophages were pretreated with EPO (as a control), or GM-CSF plus EPO, respectively. Then the two groups of “EBI-like” macrophages were co-cultured with day11 erythroblasts at a ratio of 1:20. Representative images of EBIs indicate that GM-CSF pretreated “EBI-like” macrophages surround fewer erythroblasts than control “EBI-like” macrophages (Fig. 1A). Further analyses showed that the percentages of EBIs with 3,4, or 5 or more erythroid cells significantly decrease, while the percentage of EBIs with 0 or 1–2 erythroid cells significantly increases (Fig. 1B). Previous studies have indicated that GM-CSF works by binding to its receptor for signal transduction. We checked the mRNA expression pattern of G-CSFR, GM-CSFR and M-CSFR from our published studies on EBI macrophages and the distinct stages of erythroblasts [12, 14, 17]. Interestingly, all of these three receptors are expressed by EBI macrophages (Fig. 1C). In contrast, the expression levels of G-CSFR, GM-CSFR, and M-CSFR were very low or undetectable in late-stage erythroblasts (Fig. 1C). This expression pattern was confirmed by QRT-PCR (Fig. 1D). This data suggests that GM-CSF impairs EBI formation by affecting the function of EBI macrophages but not late-stage erythroblasts. Adhesion molecules of CD163, CD169, VCAM1, EMP, and αV-integrin are significant for EBI formation [24, 27, 36–38]. To define the mechanisms of the impaired EBI formation, we examined the effects of GM-CSF on adhesion molecules expression of CD163, CD169, EMP, and αV-integrin. Figure 1E shows that GM-CSF significantly decreases the mRNA expression of CD163 but not of CD169, EMP, and αV-integrin. Taken together, this data suggests that GM-CSF impairs EBI macrophages in supporting erythropoiesis at least in part by inhibiting interaction between EBI macrophages and erythroid cells via decreased adhesion molecule expression of CD163.

### RNA-seq analysis suggests that GM-CSF treatment impairs the supporting function of human EBI macrophages during erythropoiesis

**Fig. 2:**
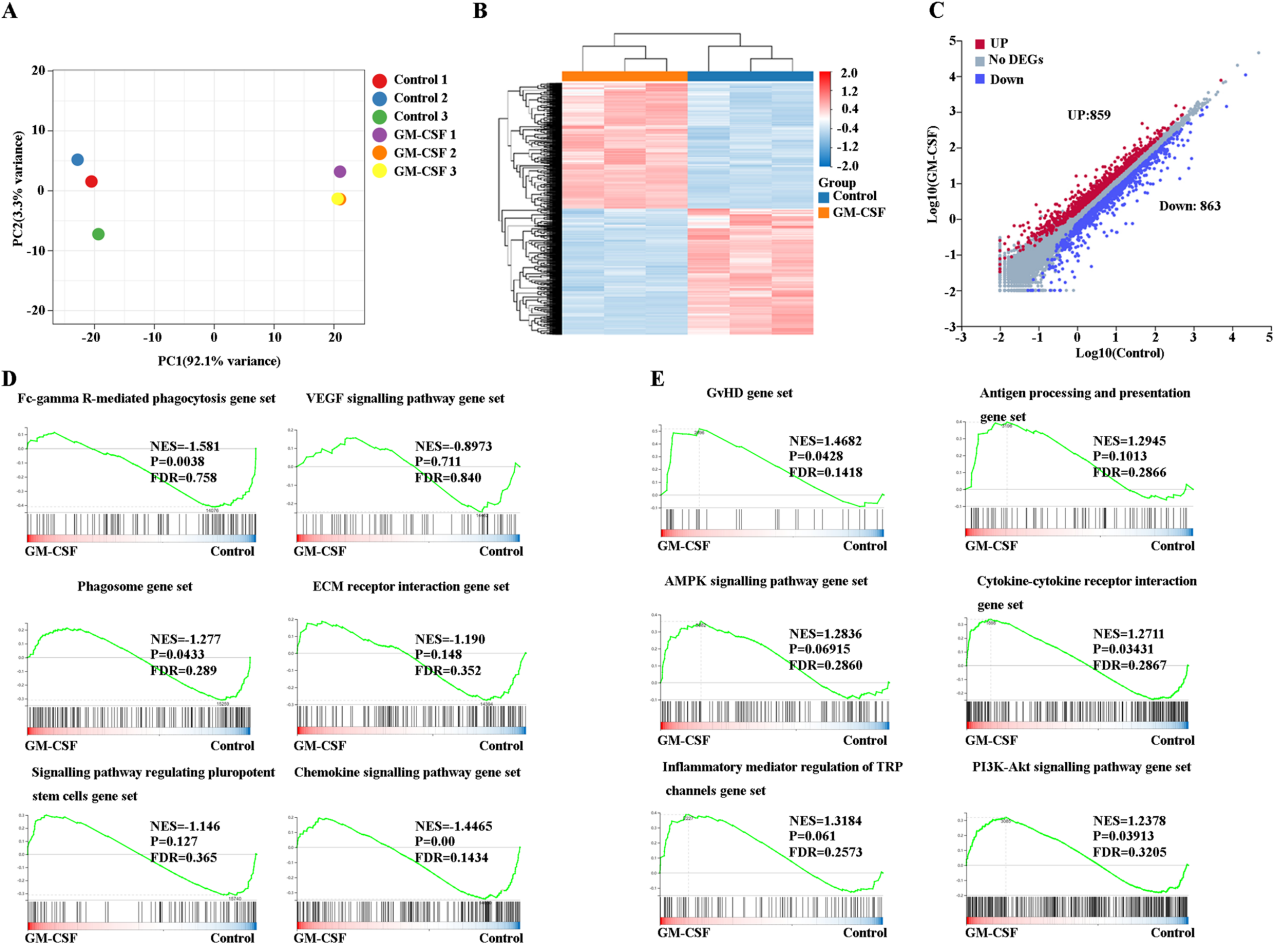
RNA-seq analysis suggests that GM-CSF treatment impairs the supporting function of human EBI macrophages during erythropoiesis. **A** Principal component analysis. **B** Heatmap of the differentially expressed genes. **C** Numbers of genes that are differentially expressed between control and GM-CSF-treated macrophages. **D** Downregulated pathways in GM-CSF-treated macrophages. **E** Upregulated pathways in GM-CSF-treated macrophages

To define transcriptional changes following GM-CSF treatment, we performed RNA-seq analysis. As expected, GM-CSF-treated human “EBI-like” macrophages clustered distinctly from control human “EBI-like” macrophages in principal component analysis (PCA; Fig. 2A). Interestingly, the analysis revealed that human “EBI-like” macrophages exposed to GM-CSF for only 24 h are already drastically distinct from control human “EBI-like” macrophages. We then performed pairwise comparison of differentially expressed genes. A heatmap of the differential expression of genes is shown in Fig. 2B. A total of 1,722 genes are differentially expressed, of which 859 are up-regulated and 863 are down-regulated in GM-CSF-treated human “EBI-like” macrophages versus control human “EBI-like” macrophages (Fig. 2C). Differentially expressed genes are listed in Additional file 12: Table S4. GSEA analysis of the differentially expressed genes revealed that the top upregulated pathways in GM-CSF-treated human “EBI-like” macrophages include Graft-versus-host disease (GvHD), inflammatory-mediator regulation of TRP channels, antigen processing and presentation, AMPK, PI3K-AKT, and cytokine-cytokine receptor interaction (Fig. 2E). In contrast, the top down-regulated pathways in GM-CSF-treated human “EBI-like” macrophages are mostly related to Fc-gamma R-mediated phagocytosis, the VEGF signaling pathway, phagosomes, ECM receptor interaction, signaling pathway regulating pluripotent stem cells, and the chemokine signaling pathway. (Fig. 2D).

**Fig. 3:**
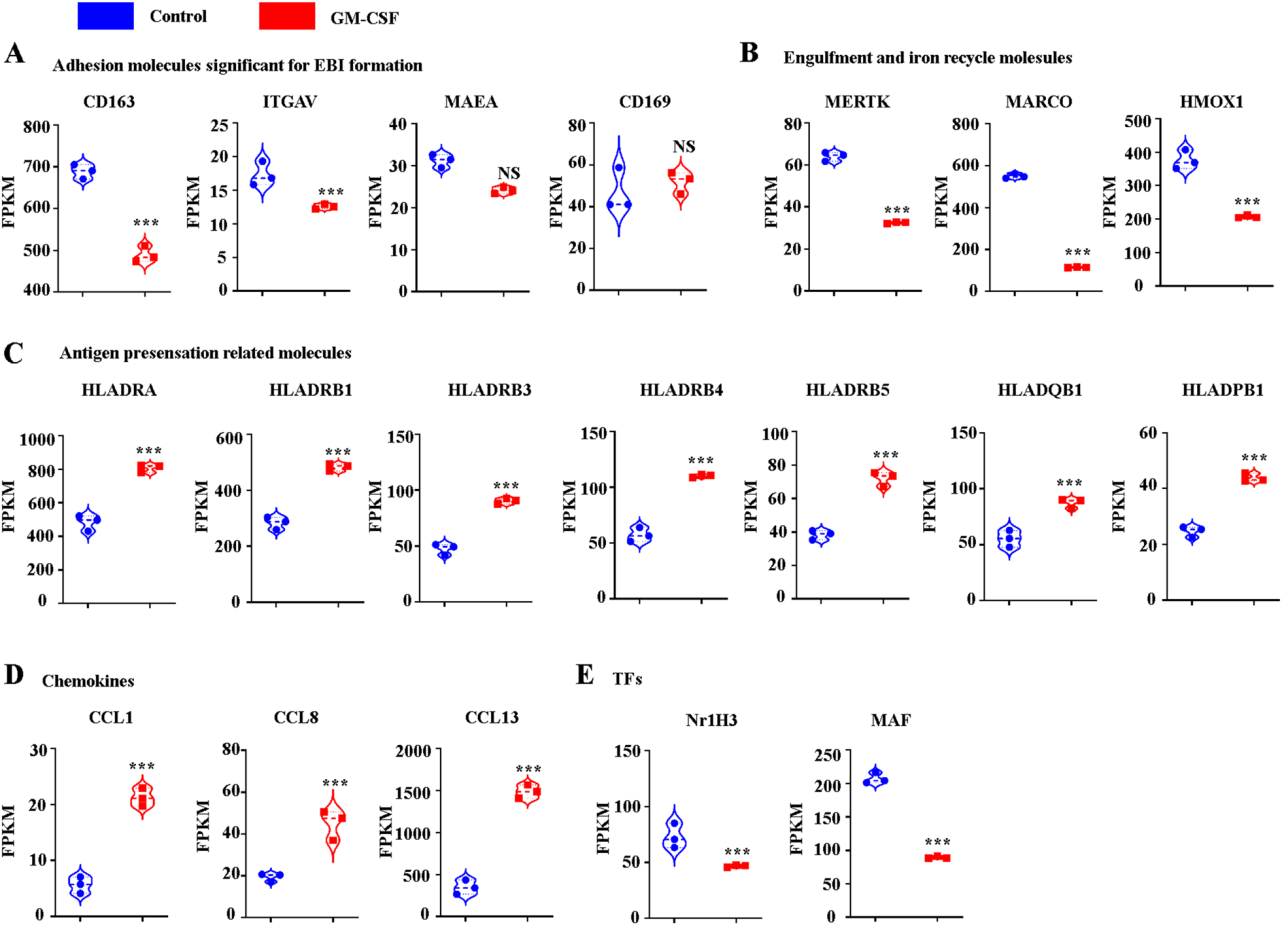
The FPKM value of several significant genes. **A** The FPKM value of adhesion molecules involved in macrophage-erythroblast interaction. **B** The FPKM value of phagocytosis associated and iron-recycling genes. **C** The FPKM value of antigen presenting genes. **D** The FPKM value of chemokines and cytokines. **E** The FPKM value of transcription factors

Our previous research indicated that the expression levels of genes encoding proteins known to be important for the EBI macrophage function of supporting erythropoiesis include adhesion molecules, molecules for nucleus engulfment and digestion, iron recycling molecules, and growth factors [17]. GM-CSF significantly decreases adhesion molecule expression of CD163 but not of CD169, while the expression of MAEA and αV-integrin (enriched in ECM receptor interaction) slightly decreases upon GM-CSF treatment (Fig. 3A). MERTK and MARCO are significant for the engulfment of EBI macrophages, while GM-CSF treatment decreases the expression of MERTK and MARCO (Fig. 3B). HMOX1 is important for iron recycling, while GM-CSF treatment decreases the expression of HMOX1 (Fig. 3B). IGF1, IL-18, and VEGF-B are the main growth factors secreted by EBI macrophages for erythroblast proliferation, while there are no significant differences between control and GM-CSF-treated EBI macrophages (data not shown). Overall, GM-CSF impairs EBI macrophage functioning by decreasing adhesion between erythroblasts and EBI macrophages, decreasing engulfment, and decreasing iron recycling.

### RNA-seq analysis suggests that GM-CSF treatment upregulates the immune regulatory function of EBI macrophages

Our and other groups’ findings indicate that EBI macrophages are M2-like macrophages that support erythropoiesis via multiple mechanisms [17, 39]. In contrast, GM-CSF mainly stimulates the diverse functions of macrophages, including induction of MHC-class II and pattern recognition receptor (PRR) expression, antigen processing and presentation, cell adhesion and chemotaxis for leukocytes, migration for leukocytes, and so on [40, 41]. In the present study, we described that the GvHD pathway, antigen processing and presentation, and inflammatory-mediator regulation of TRP channels are enriched with GM-CSF treatment. Interestingly, of the enriched gene sets, expression of HLA-DRA, HLA-DRB1,3,4,5, HLA-DQB1, HLA-DPB1, and CD83 significantly increased upon GM-CSF treatment (Fig. 3C). HLA-DRA, HLA-DRB, HLA-DQB, and HLA-DPB are the families of the HLA class, which plays a central role in the immune system and immune response by presenting peptides derived from extracellular proteins, in particular, pathogen-derived peptides to T cells. Importantly, the GvHD pathway, antigen processing and presentation, and inflammatory-mediator regulation of TRP channels enriched in GM-CSF treatment all include the HLA-DRA, HLA-DRB1,3,4,5, HLA-DQB1, and HLA-DPB genes. Additionally, C–C motif chemokine ligand 1(CCL1), CCL8 and CCL13 are involved in immunoregulatory and inflammatory processes that also increase after GM-CSF treatment, suggesting the immune regulatory function of GM-CSF treated “EBI-like” macrophages (Fig. 3D). Thus, GM-CSF treatment upregulates the immune regulatory function of EBI macrophages.

### GM-CSF treatment decreases the key transcription factors MAF and NR1H3.

Gene expression is regulated by transcription factors. MAF and NR1H3 are the two main selective transcription factors (TFs) expressed by EBI macrophages. ChIP-X Enrichment Analysis Version 3 (ChEA3) indicates that several key molecules significant for the function of EBI macrophages such as CD163, VCAM1, HMOX1,MERTK,AXL, and IGF1 may be regulated by NR1H3 or MAF (Additional file 1: Figure S1; [42]). We then analyzed the differentially expressed TFs between control and GM-CSF-treated EBI macrophages. Interestingly, MAF and NR1H3 dramatically decreased upon GM-CSF treatment (Fig. 3E). Collectively, GM-CSF induced many gene changes that may be at least partially correlated with down-regulation of MAF and NR1H3.

### GM-CSF treatment leads to decreased erythroblasts and EBI formation in mouse BM

**Fig. 4:**
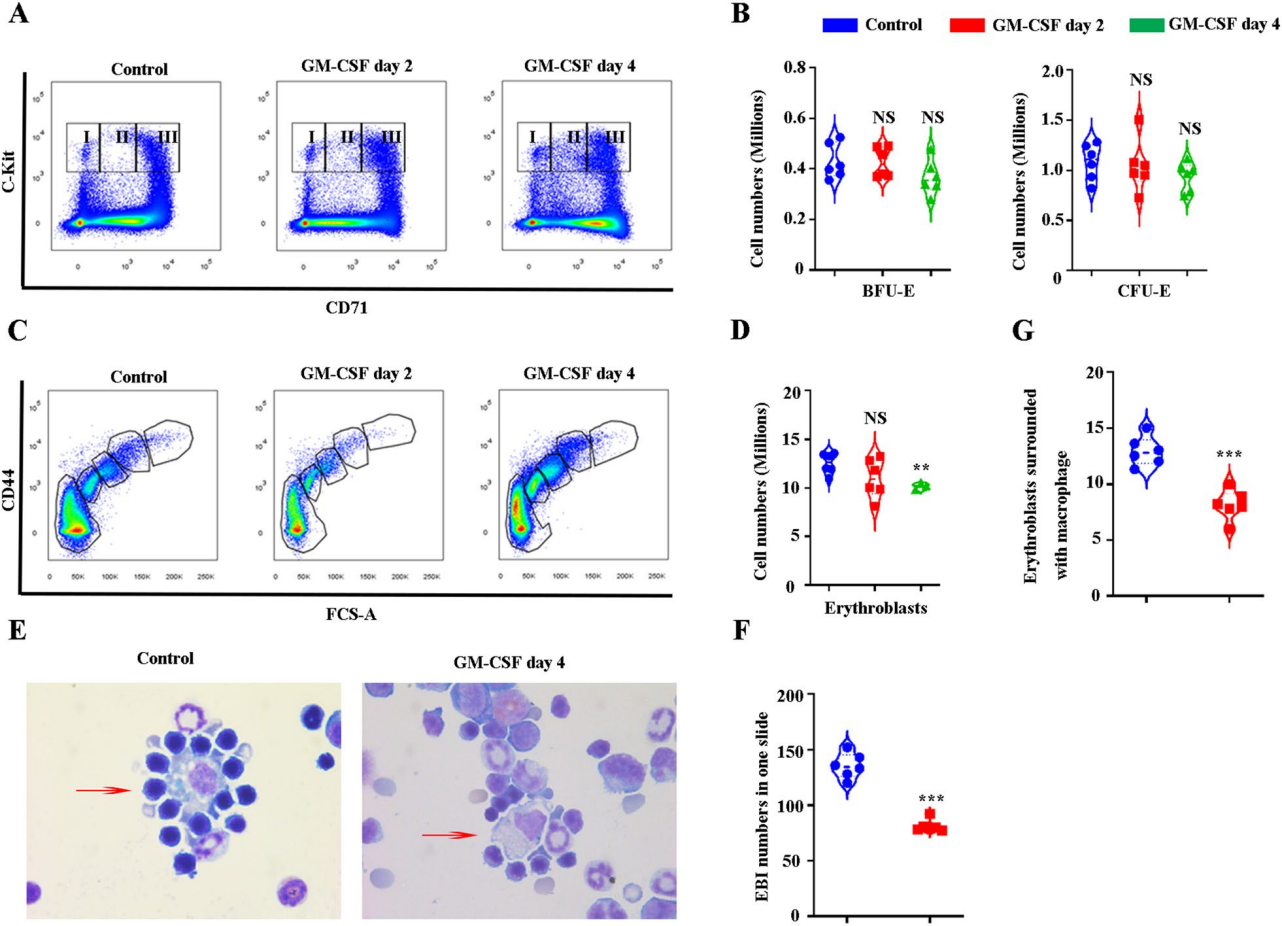
GM-CSF treatment leads to decreased erythroblasts and EBI formation in mouse BM. **A** Plot of CD71 versus c-Kit of DAPI^−^Linages^−^CD16^−^CD32^−^CD41^−^CD34^−^Sca1^−^cells in control and GM-CSF-treated mice BM (I: BFU-E, II: BFU-E/CFU-E mixture and III: CFU-E). **B** Quantitative analyses of BFU-E and CFU-E numbers in control and GM-CSF-treated mice BM. **C** Representative plot of CD44 versus FSC of Ter119^+^cells revealing various staged erythroid cells in control and GM-CSF-treated mice BM. **D** Quantitative analysis of erythroblasts. **E** Representative cytospin images of EBI enrichment in control and GM-CSF treated mice BM. **F** Quantitative analysis of EBI numbers in one slide. **G** Quantitative analysis of erythroblasts surrounded with macrophages. *P < 0.05, ** *P* < 0.01 *** *P* < 0.001. N = 6

Having shown that GM-CSF significantly impairs human EBI formation in vitro, we then examined how GM-CSF affect erythropoiesis in vivo. Erythropoiesis is a process by which HSCs proliferate and differentiate via multiple distinct developmental stages, to eventually generate mature RBCs. BFU-E and CFU-E are well defined as lin^−^CD16^−^CD32^−^CD41^−^CD34^−^Scal^−^CD117^+^CD71^low^ and lin^−^CD16^−^CD32^−^CD41^−^CD34^−^Scal^−^CD117^+^CD71^high^, respectively, by *Harvey F. Lodish’s* group [43]. We first stained control and GM-CSF-treated mouse BM cells and quantified BFU-E and CFU-E numbers. The gating strategy is shown in Additional file 2: Figure S2. Within the lin^−^CD16^−^CD32^−^CD41^−^CD34^−^Scal^−^ population, three populations are gated as I (CD117^+^CD71^−^), II (CD117^+^CD71^low^/^medi^), and III (CD117^+^CD71^high^, Fig. 4A). Quantitative analysis indicated that GM-CSF treatment does not affect the numbers of either BFU-E (I) and CFU-E cells (III) in mouse BM (Fig. 4B). Terminal erythroid differentiation was defined by *Xiuli An’s* group using Ter119 as the erythroid lineage marker in conjunction with CD44 and FSC [44]. The gating strategy is shown in Additional file 3: Figure S3. Using this method, erythroblast populations are clearly separated and named as Pro, Baso, Poly, and Ortho (Fig. 4C). Figure 4C depicts four populations of control and GM-CSF-treated mouse BM. Quantitative analysis indicated that GM-CSF treatment significantly decreases erythroblast numbers (Fig. 4D). EBI formation is significant for erythropoiesis [13, 17]. To further examine EBI formation under GM-CSF treatment in vivo, we enriched EBI in both control and GM-CSF-treated mouse BM. Figure 4E showed representative EBI images of control and GM-CSF treatment. Quantitative analysis demonstrated that GM-CSF significantly decreases both EBI numbers (Fig. 4F) as well as erythroblast numbers associated with EBI macrophages (Fig. 4G). Collectively, GM-CSF treatment leads to decreased erythroblast numbers and EBI formation in mouse BM. Despite a reduction in the number of BM erythroblasts, EBI macrophages, and EBI, GM-CSF treatment did not contribute to development of an apparent peripheral blood anemia (Additional file 4: Figure S4A–D).

### GM-CSF does not induce stress erythropoiesis in SP

Stress erythropoiesis is characterized by increased numbers of erythroblasts in mouse SP. We therefore analyzed the erythropoiesis in mouse SP upon GM-CSF treatment. Firstly, we examined the SP index in control and GM-CSF-treated mice. Interestingly, the SP index does not change significantly following GM-CSF treatment (Additional file 5: Figure S5A). We then stained total SP cells to analyze terminal erythroid differentiation. Additional file 5: Figure S5B presents the representative flowcytometry images, which suggest that SP is still a non-erythropoietic organ upon GM-CSF treatment. The HE staining image using frozen control and GM-CSF-treated mouse SP slices reveal no significant difference, which confirms the flowcytometry data (Additional file 5: Figure S5C and D). Hence, GM-CSF does not induce stress erythropoiesis in SP.

### GM-CSF treatment leads to decreased mouse BM EBI formation via decreased EBI macrophage numbers and CD163 and Vcam1 adhesion molecule expression

**Fig. 5:**
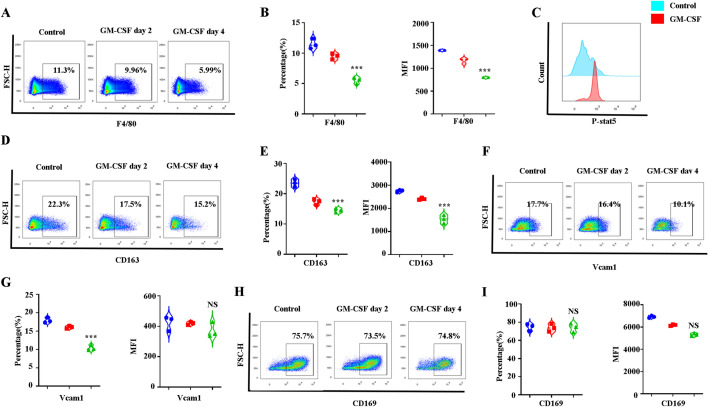
GM-CSF treatment leads to decreased mouse BM EBI formation via decreased CD163 and Vcam1 expression. **A** Plot of F4/80 versus FSC-H of DAPI^−^CD11b^−^Gr1^−^ cells in control and GM-CSF-treated mice BM. **B** Quantitative analysis of F4/80^+^ percentage and the fluorescence intensity of F4/80. **C** P-stat5 expression examined by flowcytometry after GM-CSF stimulation. **D** Plot of CD163 versus FSC-H of DAPI^−^CD11b^−^Gr1^−^ F4/80^+^macrophages in control and GM-CSF treated mice BM. **E** Quantitative analysis of CD163^+^ percentage and the fluorescence intensity of CD163. **F** Plot of Vcam1 versus FSC-H of DAPI^−^CD11b^−^Gr1^−^ F4/80^+^macrophages in control and GM-CSF-treated mice BM. **G** Quantitative analysis of Vcam1^+^ percentage and the fluorescence intensity of Vcam1. **H** Plot of CD169 versus FSC-H of DAPI^−^CD11b^−^Gr1^−^ F4/80^+^macrophages in control and GM-CSF-treated mice BM. **I** Quantitative analysis of CD169^+^ percentage and the fluorescence intensity of CD169. *P < 0.05, ** *P* < 0.01 *** *P* < 0.001. N = 3–6

To gain further insight into inhibited erythropoiesis in mouse BM upon GM-CSF administration, we analyzed EBI formation in mouse BM. EBI formation is dependent on the interaction of EBI macrophages and erythroblasts. We then examined the numbers of EBI macrophages. We stained control and GM-CSF-treated mouse BM cells with Gr1, CD11b, and F4/80. The gating strategy is shown in Additional file 6: Figure S6. Figures 5A and 5B indicate that GM-CSF treatment significantly decreases the numbers of EBI macrophages and the expression of F4/80. GM-CSF binds to GM-CSFR on macrophages to induce STAT5 phosphorylation for signaling transduction and then affects macrophage functioning. We then examined GM-CSF/GM-CSFR signaling in BM macrophages in both the control and GM-CSF-treated samples. Flowcytometry analysis revealed that GM-CSF significantly increases Stat5 phosphorylation of BM macrophages (Fig. 5C). Adhesion molecules CD163, CD169, and Vcam1 are significant for EBI formation in mouse BM. We then analyzed the CD163, CD169 and Vcam1 adhesion molecules expressed by EBI macrophages from control and GM-CSF-treated groups. Interestingly, the expression of CD163 and Vcam1 but not of CD169 significantly decreases upon GM-CSF treatment (Fig. 5D–I). Our findings demonstrate that GM-CSF impairs EBI formation at least in part by decreasing the interaction between EBI macrophages and erythroid cells via decreased EBI macrophage numbers and CD163 and Vcam1 surface expression.

### Macrophage depletion with clodronate-loaded liposomes leads to decreased erythroblast numbers in BM

Having shown that GM-CSF treatment leads to decreased erythroblast numbers in BM as well as decreased the numbers of EBI macrophages. We then examined whether erythroblast numbers in BM can be affected following macrophage depletion with clodronate-loaded liposomes. Consistent with previous studies, clodronate-loaded liposomes induced total macrophages depletion in mouse [27, 45]. Additional file 7: Figure S7A and B showed that erythroblast numbers significantly decrease in clodronate-loaded liposomes treated mouse BM. Collectively, decreased erythroblast numbers in mouse BM under GM-CSF treatment may be due to reduction in the numbers of EBI macrophages at least in part.

### GM-CSF treatment leads to decreased expression of Mertk, Axl, and Timd4 on mouse BM EBI macrophages as well as phagocytosis of senescent RBCs

**Fig. 6:**
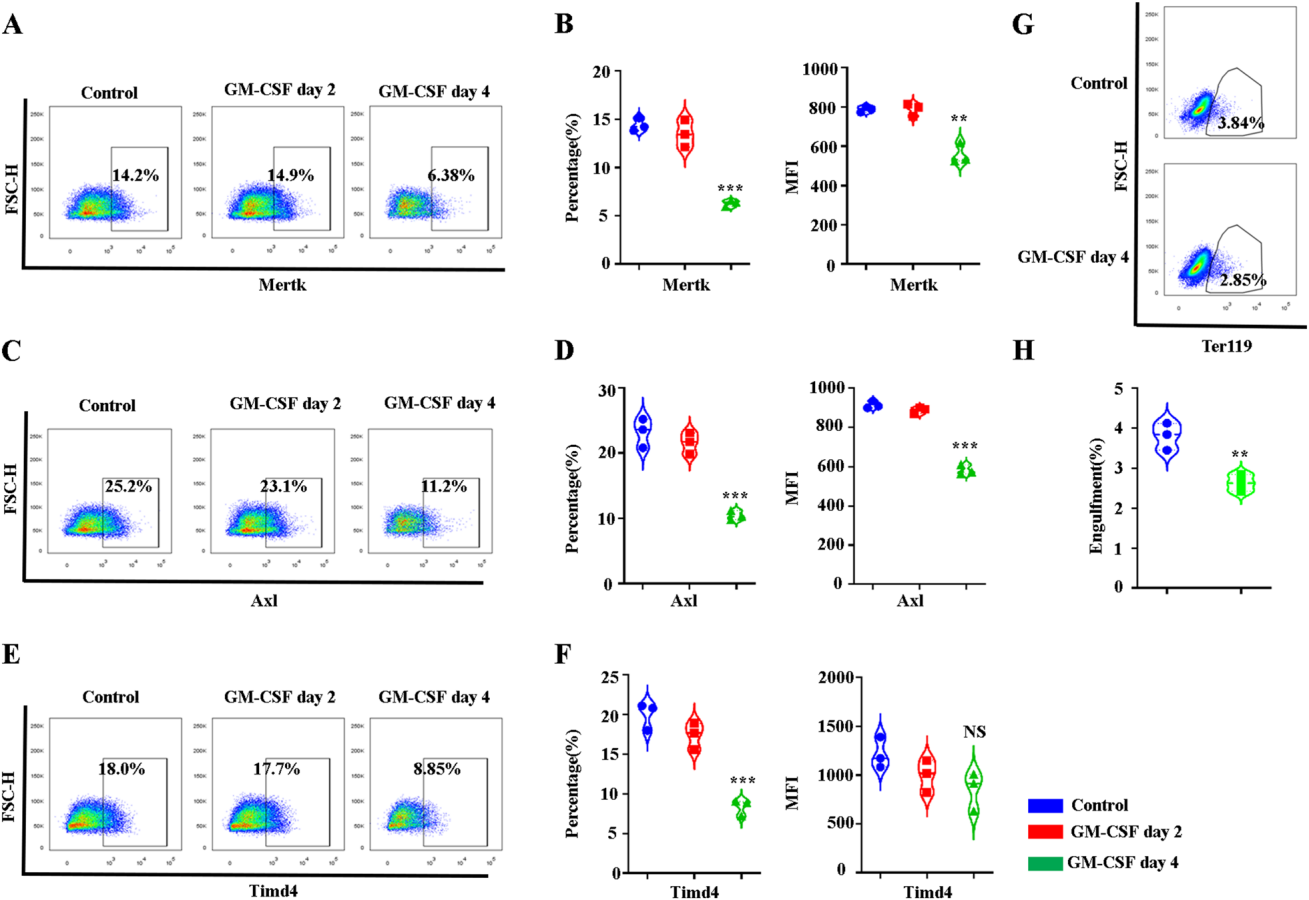
GM-CSF treatment leads to decreased expression of Mertk, Axl and Timd4 on mouse BM EBI macrophages. **A** Plot of Mertk versus FSC-H of DAPI^−^CD11b^−^Gr1^−^ F4/80^+^macrophages in control and GM-CSF treated mice BM. **B** Quantitative analysis of Mertk^+^ percentage and the fluorescence intensity of Mertk. **C** Plot of Axl versus FSC-H of DAPI^−^CD11b^−^Gr1^−^ F4/80^+^macrophages in control and GM-CSF treated mice BM. **D** Quantitative analysis of Axl^+^ percentage and the fluorescence intensity of Axl. **E** Plot of Timd4 versus FSC-H of DAPI^−^CD11b^−^Gr1^−^ F4/80^+^macrophages in control and GM-CSF-treated mice BM. **F** Quantitative analysis of Timd4^+^ percentage and fluorescence intensity of Timd4. **G** Plot of Ter119 versus FSC-H of CD11b^−^Gr1^−^ F4/80^+^macrophages in control and GM-CSF-treated mice BM. **H** Quantitative analysis of engulfment of Ter119^+^ Red blood cells of CD11b^−^Gr1^−^ F4/80^+^macrophages in control and GM-CSF treated mice BM. *P < 0.05, ** *P* < 0.01 *** *P* < 0.001. N = 3–6

Having shown that GM-CSF treatment significantly decreases the expression of MERTK on human EBI-like macrophages in vitro, we also examined the expression of Mertk, Axl, and Timd4 on mouse BM EBI macrophages in vivo. Consistent with the in vitro study, GM-CSF treatment significantly decreased the expression of Mertk, Timd4 and Axl on mouse BM EBI macrophages (Fig. 6A–F). These data indicated that GM-CSF may impair the phagocytosis of EBI macrophages during mouse BM erythropoiesis. To examine whether GM-CSF decreases phagocytosis of senescent RBCs by EBI macrophages in vivo, we performed a phagocytosis assay. Phagocytosis of senescent RBCs was defined by *Jessica A. Hamerman’s* group [28]. Among Gr1^−^CD11b^−^ F4/80^+^ macrophages that had internalized RBCs (as determined via intracellular anti-Ter119 staining, Fig. 6G), compared with control mice, GM-CSF-treated mice showed decreased Ter119^+^ percentages (Fig. 6H), suggesting decreased phagocytosis in senescent RBCs among GM-CSF-treated mice versus control mice.

### GM-CSF treatment leads to the polarization of BM EBI macrophages from M2-like to M1-like phenotype

**Fig. 7:**
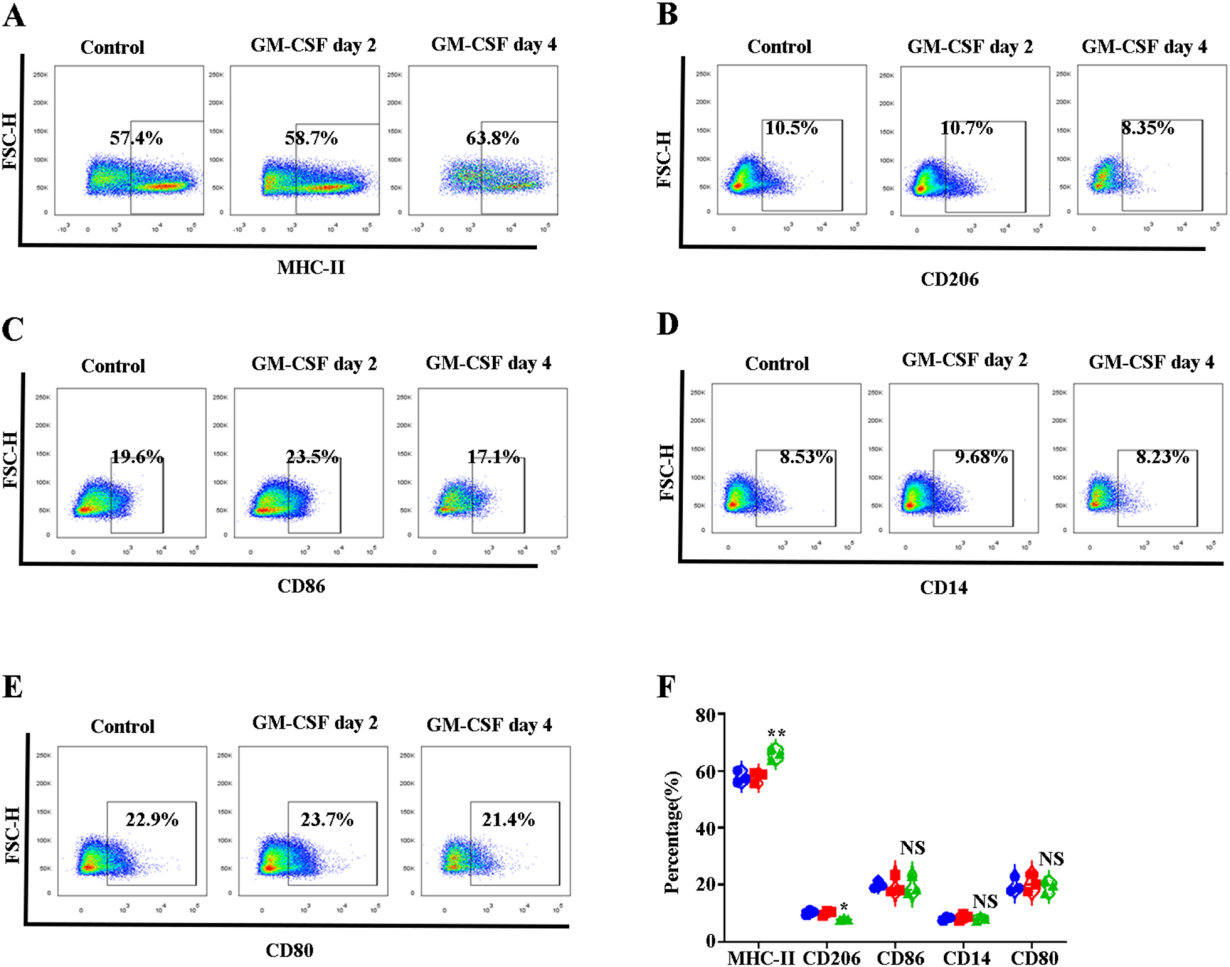
GM-CSF treatment leads to increased expression of MHC-II and decreased expression of CD206. **A** Plot of MHC-II versus FSC-H of DAPI^−^CD11b^−^Gr1^−^ F4/80^+^macrophages in control and GM-CSF-treated mice BM. **B **Plot of CD206 versus FSC-H of DAPI^−^CD11b^−^Gr1^−^ F4/80^+^macrophages in control and GM-CSF-treated mice BM. **C** Plot of CD86 versus FSC-H of DAPI^−^CD11b^−^Gr1^−^ F4/80^+^macrophages in control and GM-CSF-treated mice BM. **D** Plot of CD14 versus FSC-H of DAPI^−^CD11b^−^Gr1^−^ F4/80^+^macrophages in control and GM-CSF-treated mice BM. **E** Plot of CD80 versus FSC-H of DAPI^−^CD11b^−^Gr1^−^ F4/80^+^macrophages in control and GM-CSF-treated mice BM. **F** Quantitative analysis of MHCII^+^, CD206^+^, CD86^+^,CD14^+^,and CD80^+^ percentages

Having shown that GM-CSF treatment induces immune regulatory functions in human EBI macrophages in vitro, to examine whether GM-CSF can affect the phenotype of EBI macrophages in vivo, we performed an experiment to detect the expression of M1 (MHC-II, CD14 and CD80) and M2 (CD86, CD206, CD163) surface markers on mouse BM EBI macrophages using flowcytometry. Notably, GM-CSF significantly increased the expression of MHC-II (Fig. 7A, F) but decreased the expression of CD206 (Fig. 7B, F). In addition, GM-CSF did not affect CD86 (Fig. 7C, F), CD14 (Fig. 7D, F), or CD80 expression. (Fig. 7E, F). GM-CSF significantly decreased the expression of CD163. Previous studies have indicated that GM-CSF can induce myeloid cells to secrete several inflammatory cytokines, such as IL-1 and TNF-α. We therefore examined the expression of the M1 and M2 cytokines *iNOS* and *Arg1* in control and GM-CSF-treated BM F4/80^+^ macrophages using QRT-PCR. Additional file 8: Figure S8 reflects that the expression of *iNOS*, IL-1β, and TNF-α significantly increased. In contrast, the expression of Arg1, IL-10, and TGF-β significantly decreased. Accordingly, GM-CSF leads EBI macrophages to assume a more M1-like phenotype compared with control EBI macrophages.

## Discussion

GM-CSF is barely detectable in the peripheral blood of healthy people but significantly increases in the presence of inflammatory conditions, such as, COVID-19, SCD, and so on [2, 3, 9]. Inflammatory conditions such as infection, chronic inflammatory disorders, and hematological malignancies often cause anemia, also called AI [46, 47]. Indeed, COVID-19 patients suffer a profound decline in hemoglobin levels but show an increase in the circulation of nucleated RBCs [48]. In this study, ACE2 expression peaked during erythropoiesis and rendered erythroid progenitors vulnerable to infection by SARS-CoV-2, suggesting that SARS-CoV-2 infection directly induces stress erythropoiesis. In addition, many inflammatory cytokines and chemokines are also upregulated in SARS-CoV-2 and COVID-19 patients, including GM-CSF [49, 50]. Importantly, GM-CSF was upregulated before all other inflammatory cytokines (IL-6, TNF and IFN-β) and chemokines (CCL2, CCL7 and CCL12) that were measured, indicating that GM-CSF might be involved in the initiation of this immunopathological process [49]. Additionally, a murine model of AI is commonly induced by heat-killed Brucella abortus [10]. Importantly, IL-6, and IFN-γ were significantly increased and GM-CSF was produced in substantial amounts after Brucella infection [11]. Another study also indicated that GM-CSF is an upstream regulator of inflammatory macrophage function [51]. However, the role of GM-CSF per se in adult human and mouse erythropoiesis is unclear.

In the present study, we tested the roles of GM-CSF in human and mouse EBI formation, finding that GM-CSF significantly decreases EBI formation both in vitro and in vivo. G-CSF also impairs EBI formation in vivo [20, 21]. Conversely, previous studies have shown that G-CSF induces splenic erythropoiesis at the same time [20]. Interestingly, GM-CSF does not induce splenic erythropoiesis, suggesting different roles for GM-CSF and G-CSF in erythropoiesis. As noted, the mouse model of AI featured anemia in peripheral blood [10, 11] though, GM-CSF treatment alone does not induce severe peripheral blood anemia. Given that AI resulted in upregulation of several inflammatory cytokines [10, 11], anemia is likely associated with the combined effect of these inflammatory factors.

GM-CSFR (CSF2R) is not expressed by late-stage erythroblasts but is highly expressed in human EBI macrophages, suggesting that GM-CSF may be unable to directly signal erythroblasts. Alternatively, the low expression of GM-CSFR in early-stage erythroblasts (FPKM < 10) may be insufficient, raising questions about the indirect effects of GM-CSF on erythropoiesis in vitro and in vivo. EBI, first described by *Marcel Bessis* in 1958 [15] is composed of a central macrophage surrounded by developing erythroid cells. EBI macrophages play significant roles in erythropoiesis, especially under stress conditions [17, 19, 27, 45]. Using our well-established human EBI formation system [17], we found that GM-CSF pre-treated EBI macrophages significantly impair EBI formation. Transcriptome analysis of control and GM-CSF-treated EBI macrophages by RNA-seq demonstrated that GM-CSF significantly decreases the expression of adhesion molecule CD163. Previous studies evidenced that CD163 is significant for the interaction between erythroblasts and EBI macrophages [13, 17, 37, 39]. Significantly, CD163 promotes erythroid expansion in vitro, suggesting that it enhances the proliferation and/or survival [37]. Hence, decreased expression of CD163 at least partially contributes to impaired human EBI formation and then the inhibited erythroid survival. In addition, GM-CSF significantly increases HLA-related genes and pro-inflammatory chemokines CCL1, CCL8 and CCL13, suggesting that GM-CSF polarizes EBI macrophages into the M1-like phenotype. The GSEA analysis indicated that the upregulated pathways include inflammatory mediator regulation of TRP channels, antigen processing and presentation, GvHD and so on. The down-regulated pathway includes phagosomes, the VEGF signaling pathway, ECM receptor interaction and so on. In conclusion, our results indicated that GM-CSF impairs erythropoiesis by affecting the functioning of EBI macrophages, highlighting the potential therapeutic target of GM-CSF in AI.

Because GM-CSF can stimulate numerous inflammatory processes and increase expression of pro-inflammatory chemokines, an anti-GM-CSF strategy might have broader effects than other immunomodulatory approaches in immunosuppressive therapy [3]. Indeed, clinical trials showed that GM-CSF-targeted therapy was efficacious in patients with rheumatoid arthritis who were unresponsive to anti-TNF-α therapy [52, 53]. Furthermore, clinical trials are ongoing or planned to assess the benefits of GM-CSF-targeted therapy for COVID-19 or CAR-T- or GvHD-related cytokine release syndrome (CRS; [3, 54, 55]). Thus, our and many other studies have supported the immune overactivation of GM-CSF-based putative pathogenic roles. In summary, this data suggests that GM-CSF-targeting therapy may also be a novel option for AI.

Primitive and definitive erythropoiesis in mouse embryos requires the signal transduction of GM-CSF [8]. However, adult BM erythropoiesis is different from fetal erythropoiesis as fetal erythropoiesis is a kind of stress erythropoiesis, while adult BM erythropoiesis is a steady-state erythropoiesis [13, 56]. Yet the role of GM-CSF in adult BM erythropoiesis in vivo remains mysterious. Our study demonstrates that GM-CSF significantly decreases absolute erythroblast numbers and EBI formation. Regarding the potential mechanisms for this, GM-CSF significantly decreases the number of EBI macrophages as well as the surface expression of adhesion molecules CD163 and Vcam1 (but not CD169). Significantly, clodronate-loaded liposomes decreased erythroblast numbers in BM, which partially interpret the impaired erythropoiesis in mouse BM following GM-CSF administration. EPO-EPOR-JAK2-STAT5 signal transduction in macrophages enhances EBI formation [17], and deletion of STAT5 in macrophages impair SP erythropoiesis [19]. Meanwhile, GM-CSF also performs signal transduction through JAK2-STAT5 in macrophages [57], and GM-CSF triggers JAK2-STAT5 signal transduction in BM macrophages as well. Thus, GM-CSF produces opposite effects on EBI macrophage-regulated erythropoiesis compared with EPO.

Phagocytosis of senescent RBCs represents another important role of EBI macrophages during erythropoiesis. In general, RBC clearance is thought to occur mainly in the spleen, where senescent RBCs are phagocytosed by splenic macrophages [13]. Our previous study illustrated the iron recycling machine highly expressed by mouse BM EBI macrophages, suggesting the potential role of iron recycling for EBI macrophages in the mouse BM microenvironment [17]. EPO enhances macrophage phagocytosis of apoptotic cells and also polarizes macrophages into an M2-like phenotype [58]. However, GM-CSF-treated macrophages showed significantly decreased phagocytic capacity, and GM-CSF treatment polarized macrophages into an M1-like phenotype. Previous research has indicated that M2 macrophages have higher phagocytosis functions compared to M1 macrophages [59]. In the present study, we found that GM-CSF treatment significantly decreased the expression of Mertk, Axl and Timd4. Importantly, *Mertk*^*−/−*^ mice showed significantly decreased engulfment of pyrenocytes by EBI macrophages [60]. Macrophages from *Axl*^*−/−*^ mice showed decreased a 50% decrease in phagocytotic apoptotic cells [61]. Phosphatidylserine (PS) is expressed by apoptotic cells and nuclei expelled by matured erythroblasts. Timd4 is expressed by macrophages, which bound apoptotic cells by recognizing PS. Anti-Timd4 antibodies significantly block the engulfment of apoptotic cells by macrophages [62]. As such, Mertk, Axl, and Timd4 are significant for the phagocytosis-related function of EBI macrophages. Consistent with these findings, we discovered that the engulfment of senescent RBCs of mouse BM EBI macrophages dramatically decreases following GM-CSF treatment. This data suggests that GM-CSF treatment significantly inhibits the phagocytosis function of BM EBI macrophages by decreasing phagocytosis-associated molecule expression and polarizing macrophages into an M1-like phenotype.

Many proinflammatory cytokines (e.g., TNF-α, IFN-γ, IL-1β) are known to inhibit steady-state BM erythropoiesis [63]. Heat-killed Brucella abortus are commonly used to induce AI in mouse models and have been reported to simultaneously decrease steady-state erythropoiesis in BM and to cause stress erythropoiesis in the spleen [10, 11, 63]. Our study demonstrates that the expression of TNF-α, and IL-1β on BM macrophages also increases upon GM-CSF treatment. TNF-α and IL-1β have been shown to inhibit the proliferation and differentiation of erythroid cells in BM [64, 65] while also promoting the expansion and differentiation of stress erythroid progenitors (SEPs) in the spleen in the mouse model of AI [10, 11, 63]. However, although our data indicates that GM-CSF single injection leads to inhibition of BM steady-state erythropoiesis, it does not induce stress erythropoiesis in the spleen. Overall, these data provide new insights into the complexities of BM and spleen erythroid cells and the ways in which the unique BM and spleen microenvironment affect their maturation.

In summary, we have identified previously unknown roles of proinflammatory cytokine GM-CSF in erythropoiesis. Based on our findings, we propose that GM-CSF significantly impairs erythropoiesis by affecting the functioning of EBI macrophages. This conclusion is supported by several lines of evidence. (1) GM-CSF impairs human and mouse EBI formation by decreasing the expression of adhesion molecule CD163. (2) GM-CSF reduces mouse BM erythroblast numbers by decreasing the numbers of EBI macrophages and EBI formation. (3) GM-CSF impairs phagocytosis of senescent RBCs by decreasing the engulfment-related expression of Mertk, Axl and Timd4. Although we cannot exclude the effect of GM-CSF on erythropoiesis through influencing on other myeloid cells, our findings nonetheless provide new insights into GM-CSF’s role in impairing terminal erythroid differentiation at least in part by affecting EBI macrophages, and our findings can help better understanding of elevated GM-CSF levels of inflammatory diseases. Targeting GM-CSF or GM-CSF inhibitors might be a novel option for treating AI. This conclusion must be confirmed in mice in which the GM-CSFR gene is specifically depleted by EBI macrophages.

## Supplementary Information

**Additional file 1: Figure S1**. Targeting gene expression regulated by transcription factors analyzed using ChIP-X Enrichment Analysis Version 3 (ChEA3).

**Additional file 2: Figure S2**. The gating strategy of BFU-E and CFU-E: the gating strategy of lin-CD16-CD32CD41-CD34-Scal- cell populations.

**Additional file 3: Figure S3.** The gating strategy of terminal erythroid cells: the gating strategy of CD11b-Gr1-CD45-Ter119+ cells.

**Additional file 4: Figure 4.** Peripheral Blood Routine of control and GM-CSF-treated mice: (A) RBCs assessments of control and GM-CSF-treated mice. (B) Hemoglobin assessments of control and GM-CSF-treated mice. (C) Hematocrit assessments of control and GM-CSF-treated mice. (D) Reticulocyte assessments of control and GM-CSF-treated mice. N=6.

**Additional file 5: Figure 5.** GM-CSF does not induce stress erythropoiesis in SP. (A) The SP weight index (mg/g body weight) of control and GM-CSF-treated mice. (B) Representative flowcytometry image of terminal erythroid cells in SP in control and GM-CSF-treated mice. (C) Representative HE staining image of SP in control and GM-CSF-treated mice(20X). (D) Representative HE staining image of SP in control and GM-CSF-treated mice(100X).

**Additional file 6: Figure 6.** The gating strategy of macrophages: the gating strategy of the CD11b-Gr1-SSClow cell population.

**Additional file 7: Figure 7.** (A) Representative flowcytometry image of terminal erythroid cells in liposome-control and clodronate-loaded liposomes treated mouse BM. (B) Quantitative analysis of erythroblast numbers in in liposome-control and clodronate-loaded liposomes treated mouse BM (N = 6).

**Additional file 8: Figure 8.** GM-CSF increased the mRNA expression of IL-1β, TNF-α, and iNOS and decreased the mRNA expression of IL-10, TGF-β, and Arg1. (A) The relative mRNA expression of IL-1β, TNF-α, and iNOS among GM-CSF treatment enriched BM EBI macrophages. (B) The relative mRNA expression of IL-10, TGF-β, and Arg1 among GM-CSF treatment enriched BM EBI macrophages (N = 3).

**Additional file 9: Table S1**. The list of antibodies used in this study.

**Additional file 10: Table S2**. Preparation of EBI enrichment buffer with different concentrations.

**Additional file 11: Table S3**. The list of primers used in this study.

**Additional file 12: Table S4**. The differentially expressed genes between control and GM-CSF treated human macrophages.

## Data Availability

All data generated and materials in the study are included in the present article and supplementary data.

## Abbreviations

AI: Anemia of inflammation
BM: Bone marrow
BFU-E: Burst-forming unit erythroid
CFU-E: Colony-forming unit erythroid
CCL: C–C motif chemokine ligand
EBI: Erythroblastic island
G-CSF: Granulocyte colony-stimulating factor
GM-CSF: Granulocyte–macrophage colony-stimulating factor
GvHD: Graft-versus-host disease
HSCs: Hematopoietic stem cells
IL-6: Interleukin-6
IFN-γ: Interferon-γ
OCT: Optimal cutting temperature
PS: Phosphatidylserine
RNA-seq: RNA sequencing
SCD: Sickle cell disease
SEPs: Stress erythroid progenitors
VCAM-1: Vascular cell adhesion molecule-1
