# Supplementary figures and images for: GM-CSF impairs erythropoiesis by disrupting erythroblastic island formation via macrophages
Source: J Transl Med. 2022 Jan 3;20:11. doi: 10.1186/s12967-021-03214-5 (PMC8721478; doi:10.1186/s12967-021-03214-5)

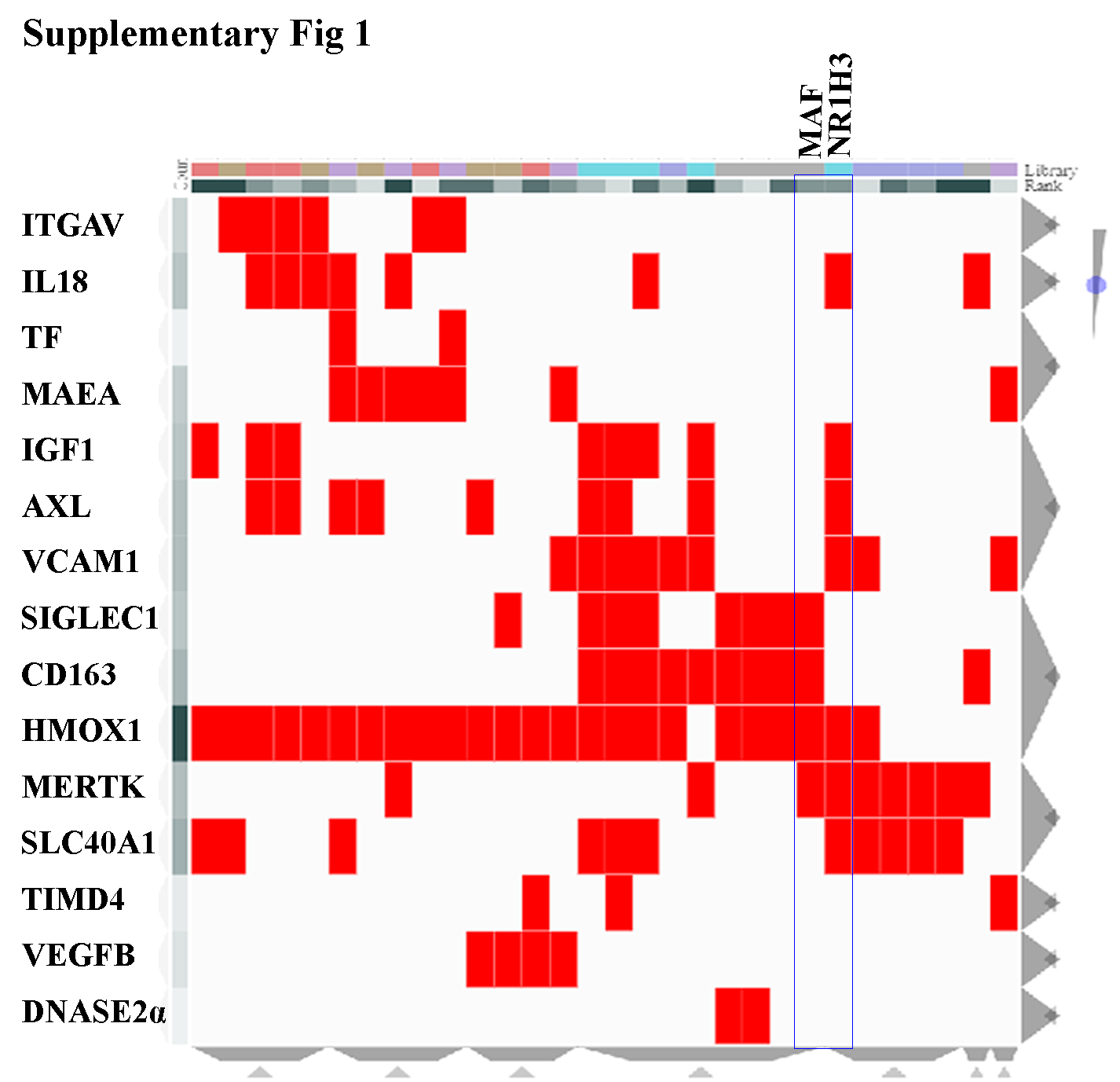

Supplement: Supplementary file 1 — Additional file 1: Figure S1. Targeting gene expression regulated by transcription factors analyzed using ChIP-X Enrichment Analysis Version 3 (ChEA3). [file 12967_2021_3214_MOESM1_ESM.tif]

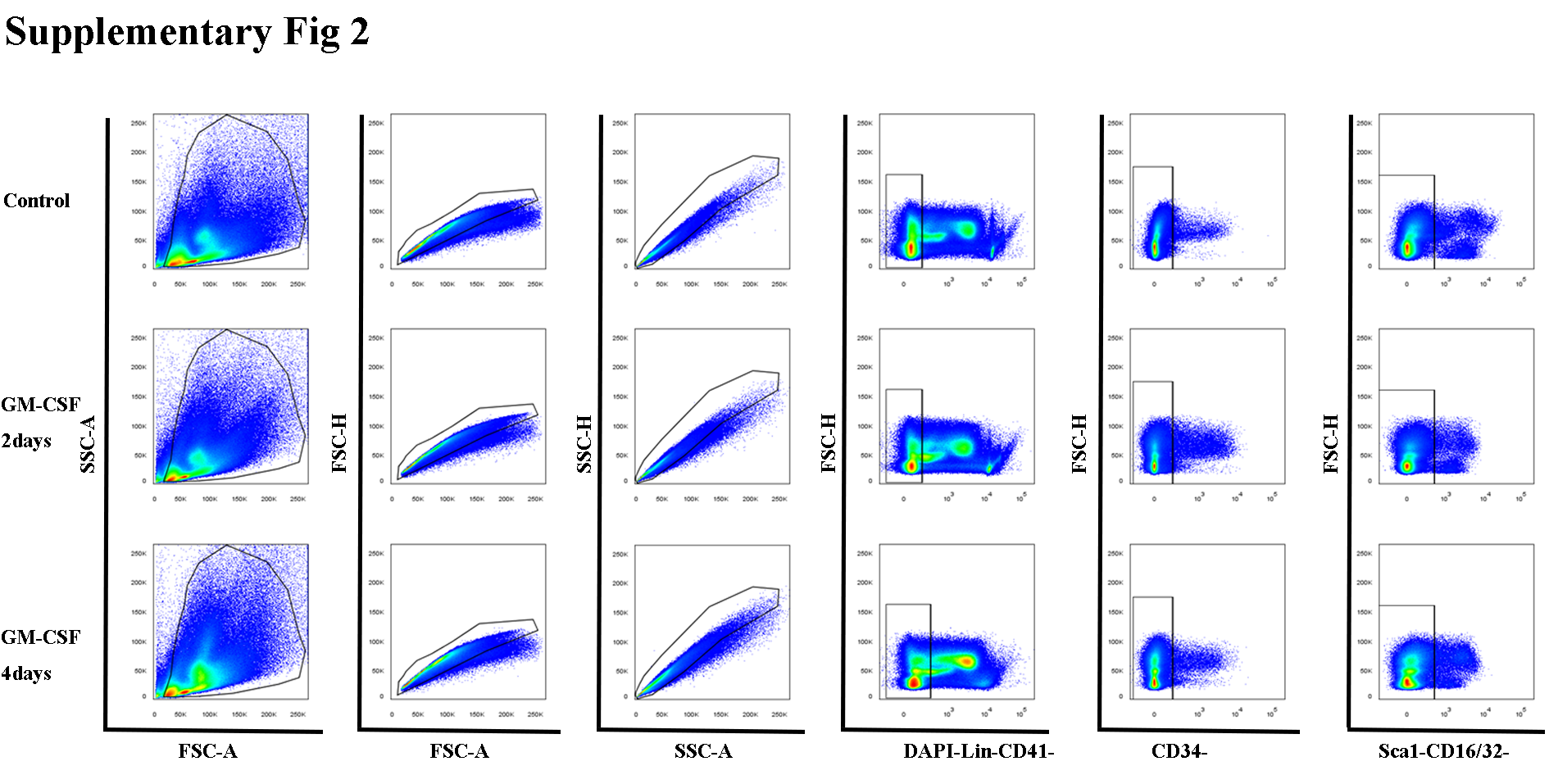

Supplement: Supplementary file 2 — Additional file 2: Figure S2. The gating strategy of BFU-E and CFU-E: the gating strategy of lin-CD16-CD32CD41-CD34-Scal- cell populations. [file 12967_2021_3214_MOESM2_ESM.tif]

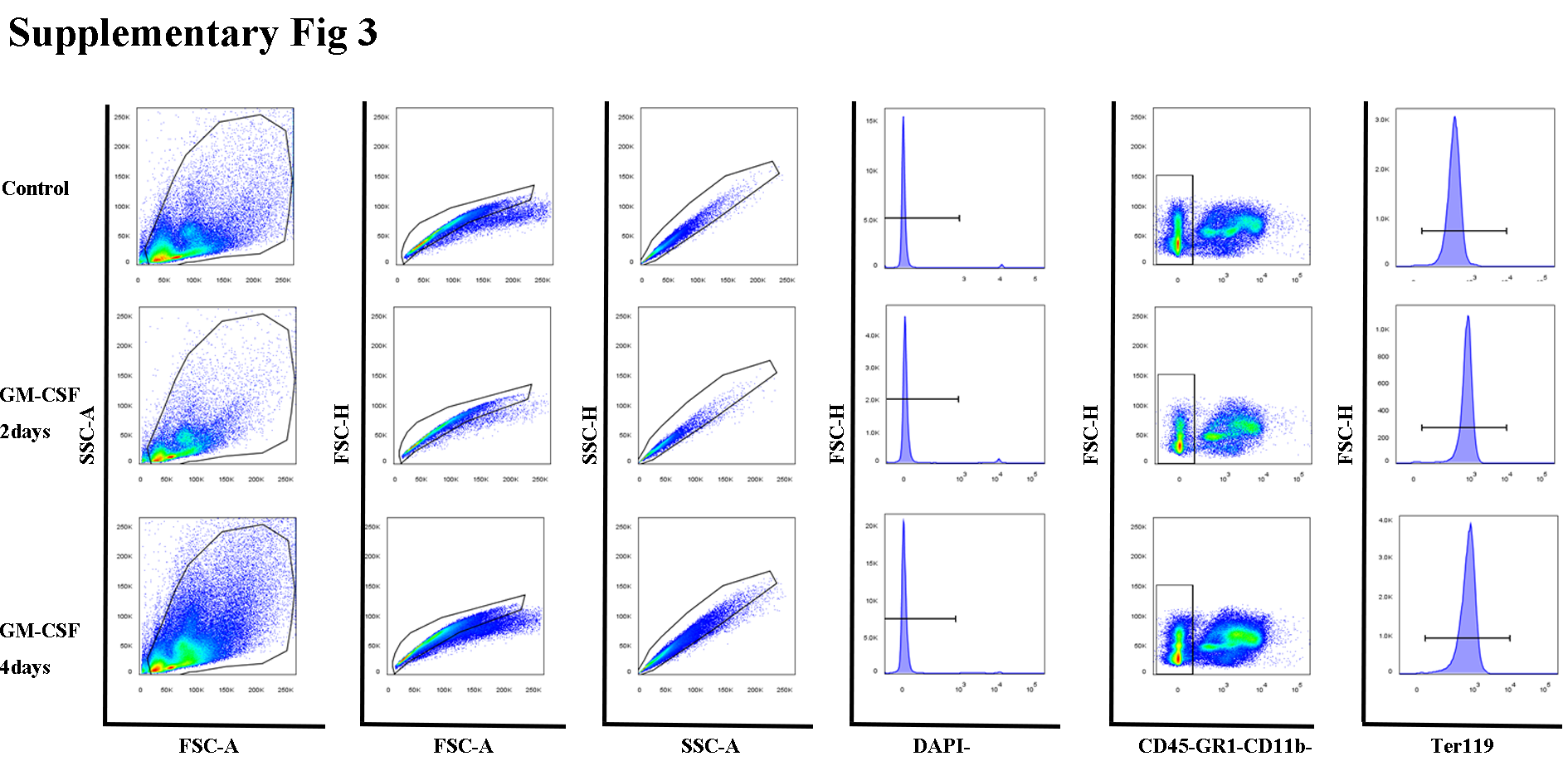

Supplement: Supplementary file 3 — Additional file 3: Figure S3. The gating strategy of terminal erythroid cells: the gating strategy of CD11b-Gr1-CD45-Ter119+ cells. [file 12967_2021_3214_MOESM3_ESM.tif]

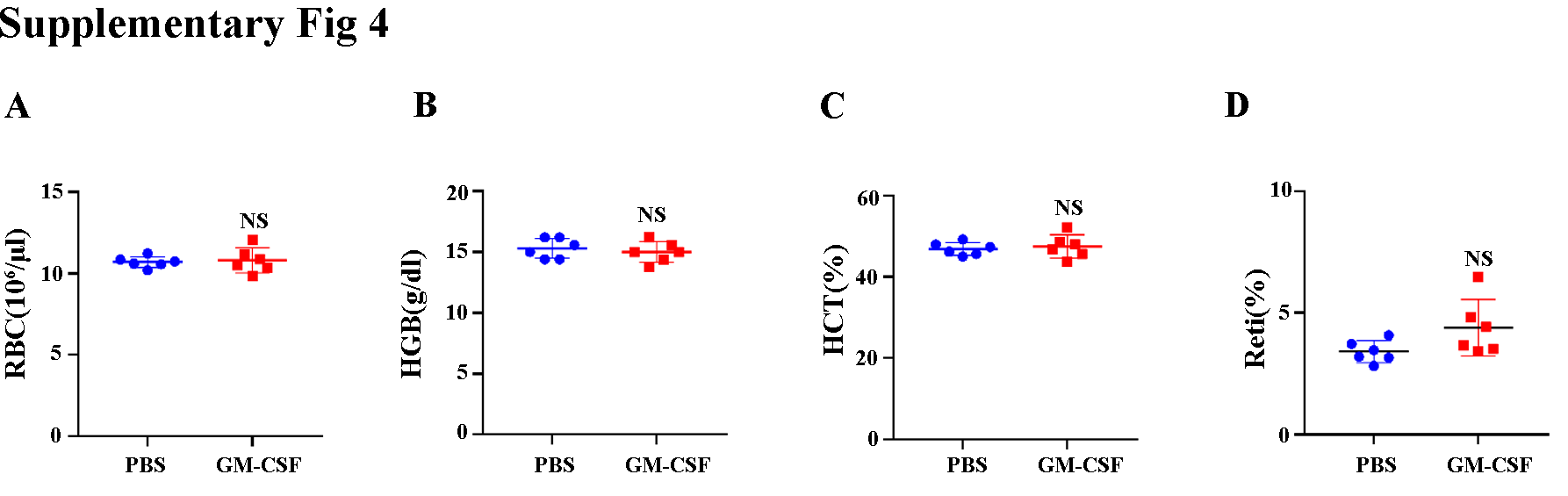

Supplement: Supplementary file 4 — Additional file 4: Figure 4. Peripheral Blood Routine of control and GM-CSF-treated mice: (A) RBCs assessments of control and GM-CSF-treated mice. (B) Hemoglobin assessments of control and GM-CSF-treated mice. (C) Hematocrit assessments of control and GM-CSF-treated mice. (D) Reticulocyte assessments of control and GM-CSF-treated mice. N=6. [file 12967_2021_3214_MOESM4_ESM.tif]

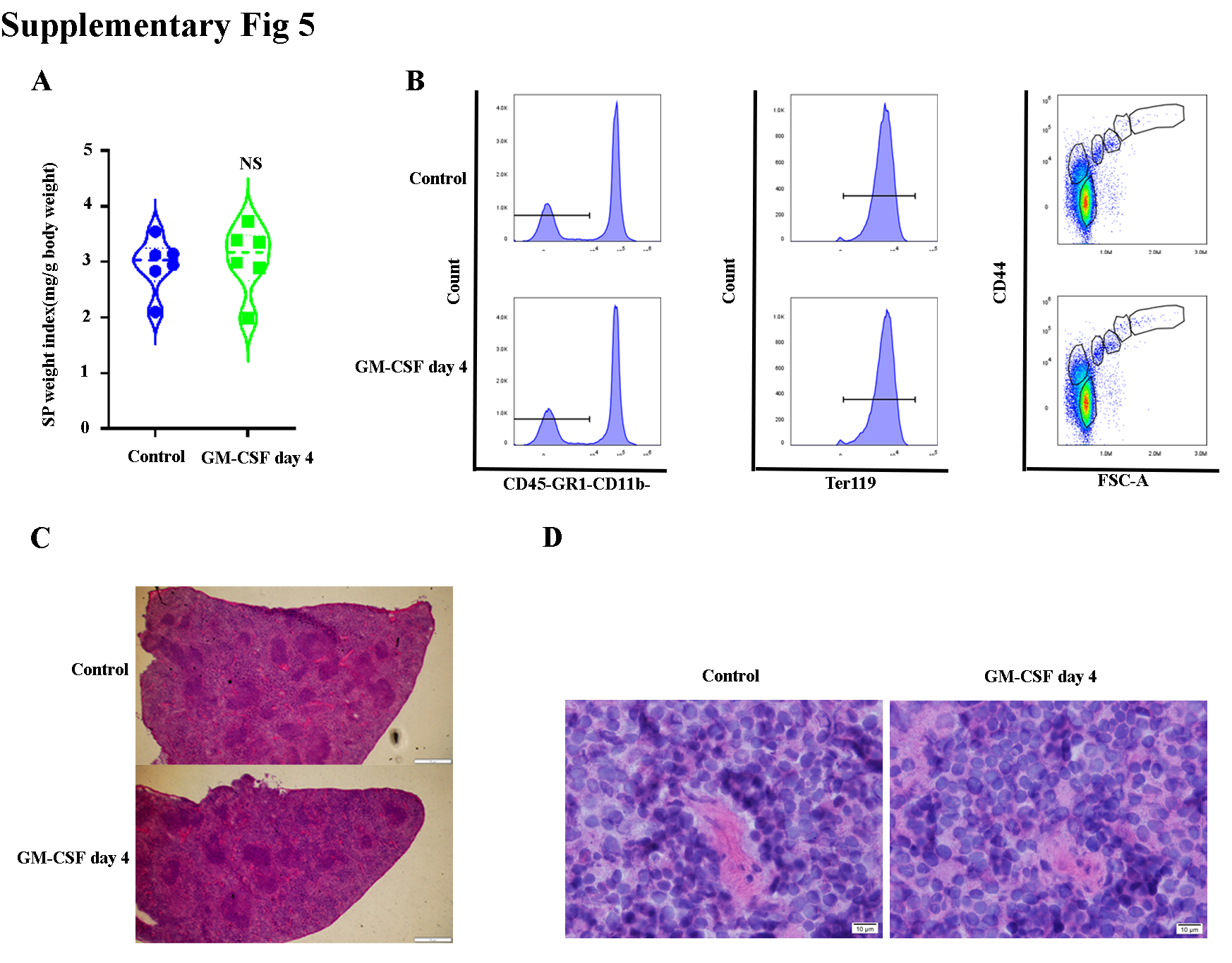

Supplement: Supplementary file 5 — Additional file 5: Figure 5. GM-CSF does not induce stress erythropoiesis in SP. (A) The SP weight index (mg/g body weight) of control and GM-CSF-treated mice. (B) Representative flowcytometry image of terminal erythroid cells in SP in control and GM-CSF-treated mice. (C) Representative HE staining image of SP in control and GM-CSF-treated mice(20X). (D) Representative HE staining image of SP in control and GM-CSF-treated mice(100X). [file 12967_2021_3214_MOESM5_ESM.tif]

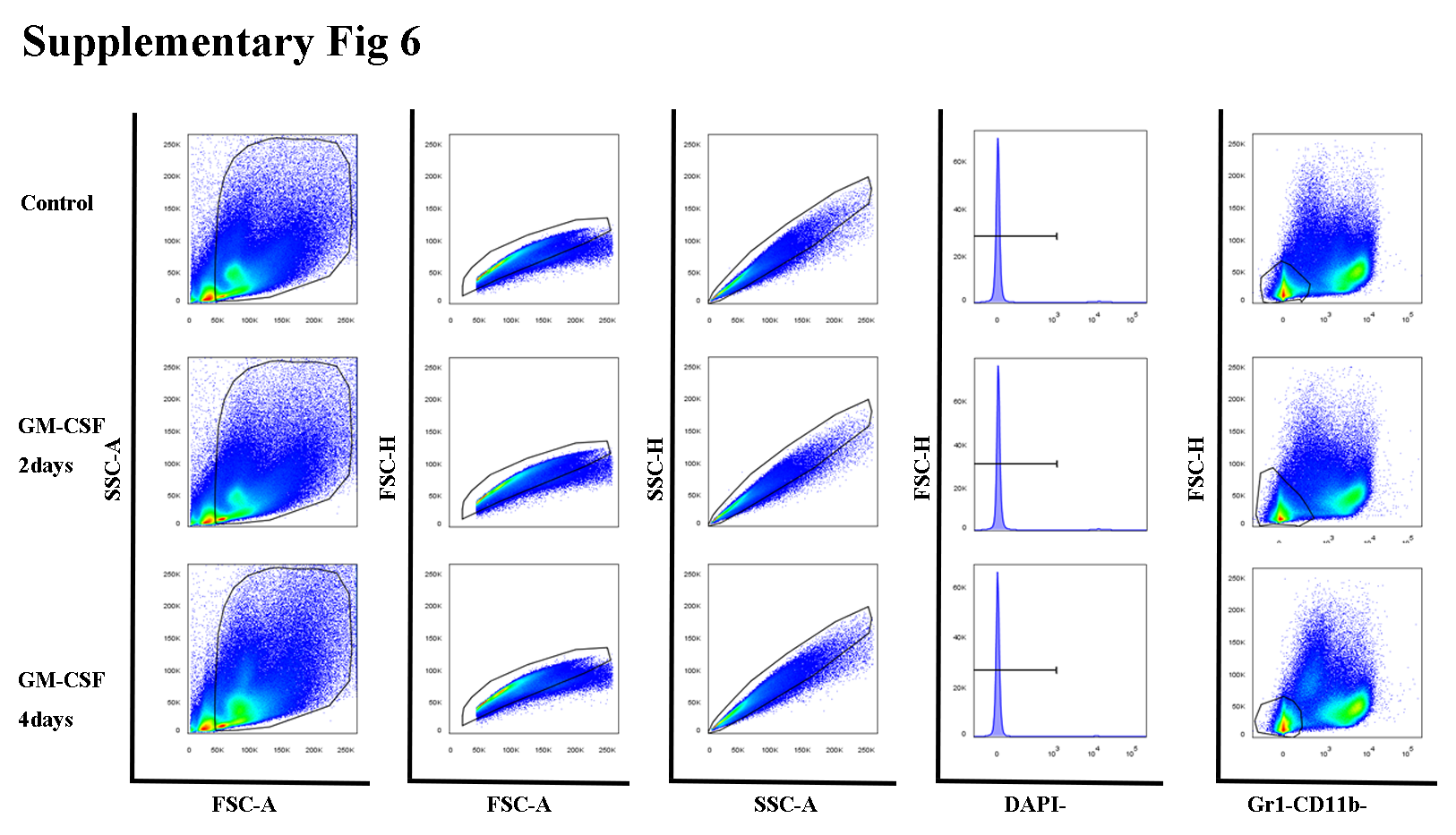

Supplement: Supplementary file 6 — Additional file 6: Figure 6. The gating strategy of macrophages: the gating strategy of the CD11b-Gr1-SSClow cell population. [file 12967_2021_3214_MOESM6_ESM.tif]

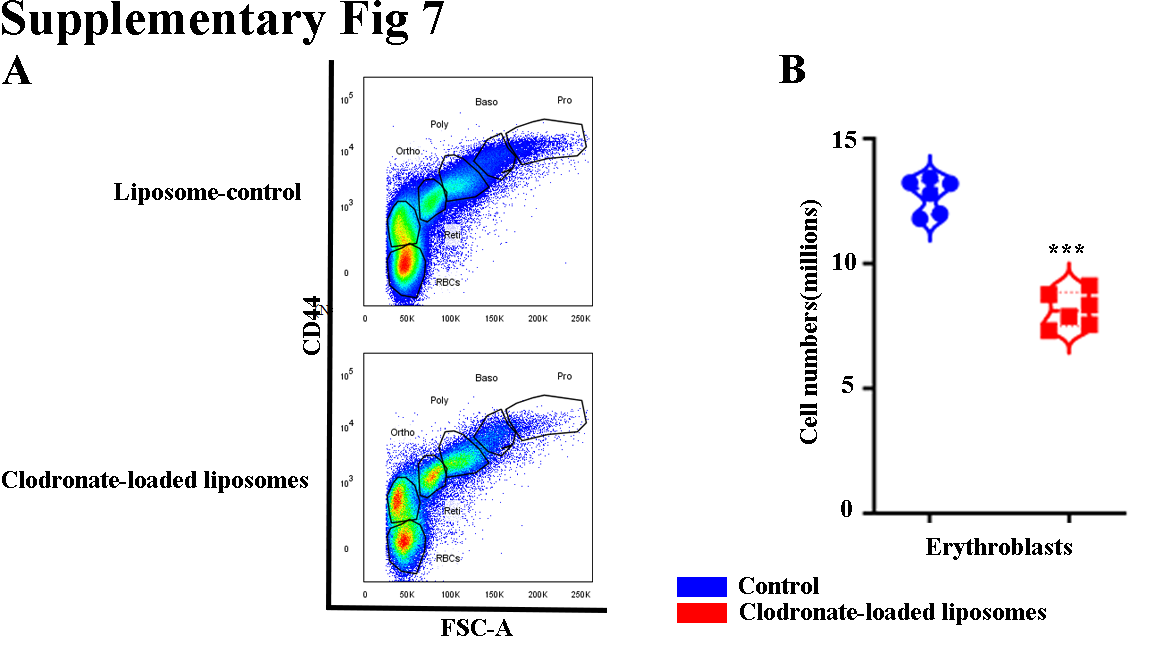

Supplement: Supplementary file 7 — Additional file 7: Figure 7. (A) Representative flowcytometry image of terminal erythroid cells in liposome-control and clodronate-loaded liposomes treated mouse BM. (B) Quantitative analysis of erythroblast numbers in in liposome-control and clodronate-loaded liposomes treated mouse BM (N = 6). [file 12967_2021_3214_MOESM7_ESM.tif]

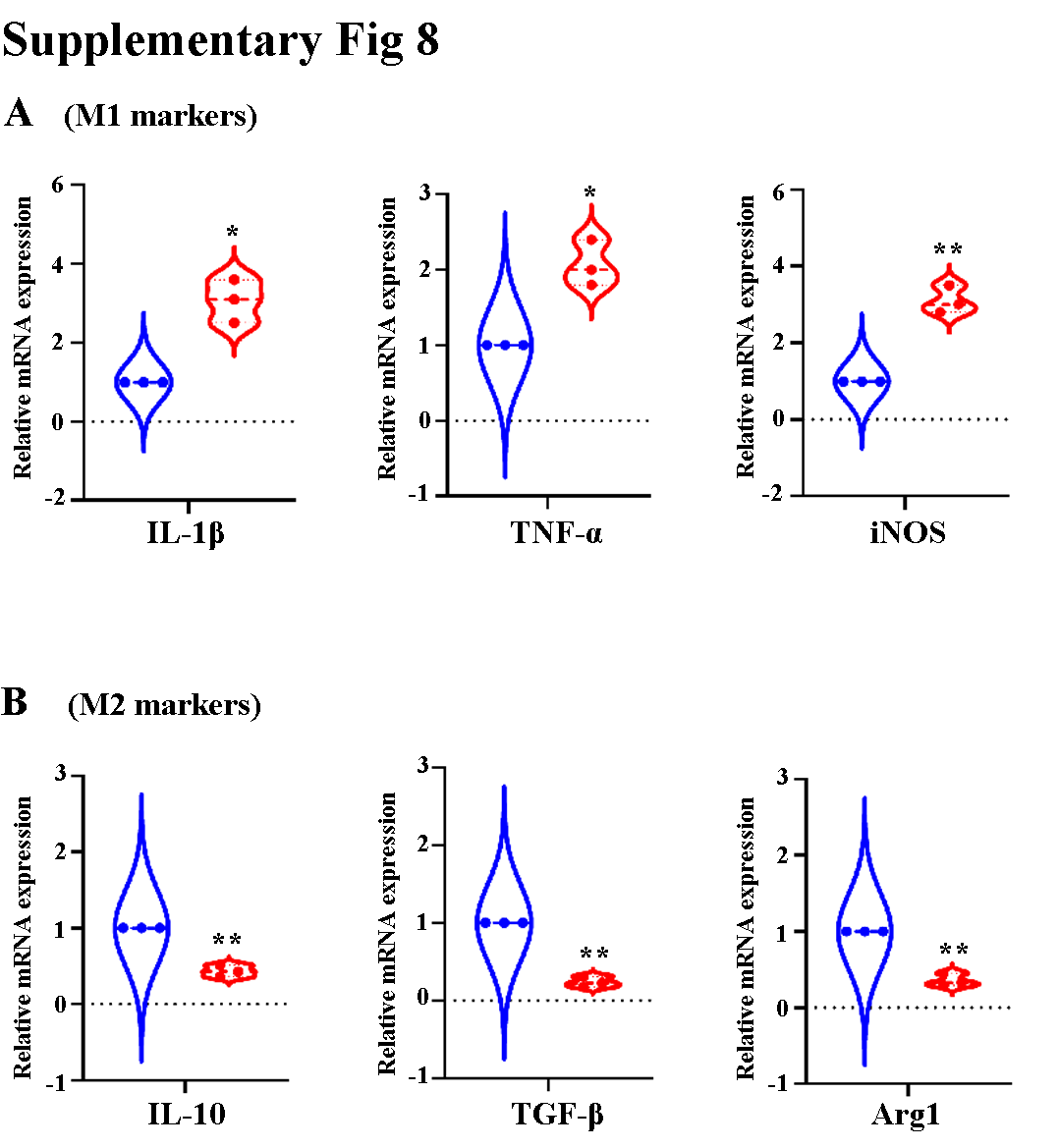

Supplement: Supplementary file 8 — Additional file 8: Figure 8. GM-CSF increased the mRNA expression of IL-1β, TNF-α, and iNOS and decreased the mRNA expression of IL-10, TGF-β, and Arg1. (A) The relative mRNA expression of IL-1β, TNF-α, and iNOS among GM-CSF treatment enriched BM EBI macrophages. (B) The relative mRNA expression of IL-10, TGF-β, and Arg1 among GM-CSF treatment enriched BM EBI macrophages (N = 3). [file 12967_2021_3214_MOESM8_ESM.tif]
